# Computational Analysis of the Kinetic Requirements for Coupled Reaction Systems

**DOI:** 10.3390/molecules30040911

**Published:** 2025-02-15

**Authors:** Sara Incarbone, Luca De Gioia

**Affiliations:** Department of Biotechnology and Biosciences, University of Milan-Bicocca, Piazza della Scienza 2, 20126 Milan, Italy

**Keywords:** computational model, endergonic synthesis, kinetics, simulation

## Abstract

The art of designing coupling systems to drive reactions for endergonic synthesis is a subject of great interest in the scientific community, but it still presents major challenges. The aim of this kinetic study was to run simulations in COPASI 4.39 to test the behavior of hypothetical models for a system that couples two independent reactions, one exergonic and the other endergonic. In our computational study, we unraveled the qualitative and quantitative conditions that allow and benefit coupling, considering all possible reaction pathways within the network. Optimal conditions were reached by assigning favorable directionalities and low activation energies to six reaction steps within a network that featured twenty reaction steps. Moreover, different models were designed and tested in order to investigate the availability of coupling with different reaction steps.

## 1. Introduction

Chemical reactions are typically governed by either thermodynamic or kinetic control [1,2]. Under thermodynamic control, the distribution of products reflects the differences in free energy among potential species, favoring the most stable thermodynamic state. In contrast, kinetic control directs the reaction toward products that form the fastest, reaching local energy minima due to lower activation energy barriers. Catalysts play a pivotal role in modulating reaction pathways, as they lower activation energies and facilitate product formation through kinetic selection—a principle that underpins processes like asymmetric catalysis.

Recently, a third paradigm for reaction control has been described: ratchet mechanisms [3,4,5]. Unlike thermodynamic and kinetic controls, ratchets leverage an independent energy flow—such as light, chemical reactions, electrochemical gradients or mechanical force—to navigate complex energy landscapes [6]. This mechanism enables access to reaction outcomes unattainable by conventional controls. Specifically, molecular ratchets exploit energy derived from spontaneous processes to drive kinetically-selected pathways. For example, the energy from an exergonic transformation can drive a coupled endergonic process, effectively enabling movement “uphill” on the energy landscape [7]. Of course, the requirement that the overall process is “downhill” ensures that the second law of thermodynamics is not violated. During catalyst turnover, the cycle operates far from equilibrium due to energy input from the substrate-to-product conversion. This disequilibrium fuels a chemical process, indirectly coupling energy sources through a molecular engine—a hallmark of ratchet mechanisms.

Biological systems exemplify the power of ratchet mechanisms, as life fundamentally depends on maintaining systems away from equilibrium [8]. At the molecular scale, biological “engines” couple thermodynamically spontaneous processes with endergonic reactions, enabling essential functions such as macromolecular synthesis. Ribosomes, DNA and RNA polymerases, fatty acid synthase, and ATP (adenosine triphosphate) synthase are archetypes of molecular machines that employ ratchet mechanisms to achieve synthesis [9]. The critical role of biological ratchets is underscored by the observation that 30–50% of a cell’s energy expenditure is dedicated to the ribosome’s synthesis, operation, and maintenance [10].

Two primary types of ratchets are relevant to chemistry and biochemistry [11,12,13,14]. The information ratchet, prominent in biomolecular systems, involves a chemical engine that catalyzes a “fuel-to-waste” reaction, such as ATP hydrolysis, which produces ADP (adenosine diphosphate) and phosphate [15,16]. This mechanism exploits substrate properties—such as position or binding affinity—to modulate reaction kinetics. Synthetic analogues of information ratchets, powered by chemical or photonic energy, have recently been developed to perform work through similar principles. The second type, the energy ratchet, achieves directional bias through periodic or stochastic switching between energy surfaces, altering the relative depths of energy minima and heights of maxima [4]. This enables statistically-directed transport across the potential energy landscape independent of substrate-specific interactions. Energy ratchets are often driven by stepwise processes fueled by diverse energy inputs, including light, chemical gradients, and mechanical force.

Inspiration from biological systems highlights the utility of information ratchets, which perform work by kinetically selecting pathways based on the chemical state of the ratchet within its cycle. For synthetic design, chemically-fueled information ratchets aim to achieve directionality through asymmetric transition-state barriers that favor the desired reaction pathway [3,17,18]. This principle allows for the coupling of reactions to drive thermodynamically unfavorable (endergonic) processes in a targeted manner.

**Figure 1:**
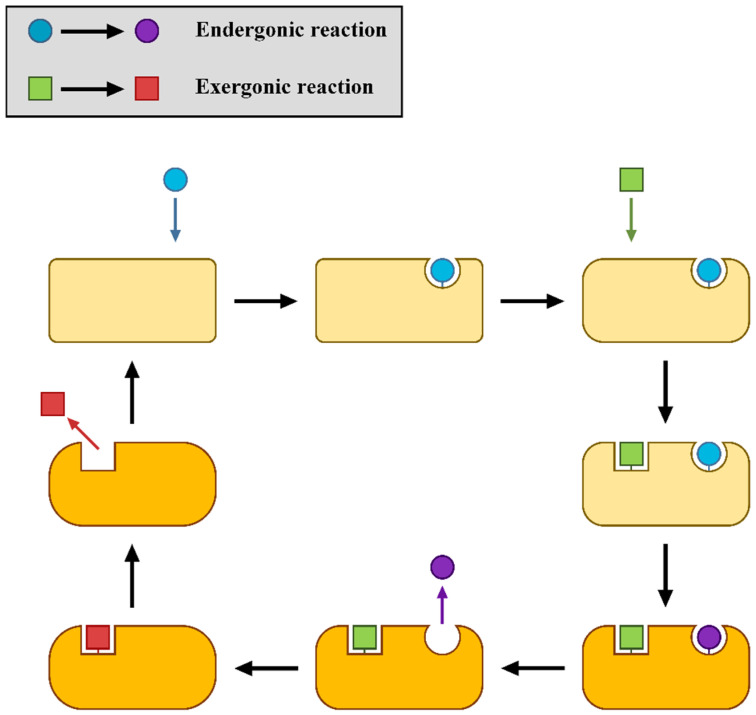
Representation of a possible coupling system featuring the different states of the molecular system that allows the coupling. In its starting state (upper left), the system can only bind the reagent of the endergonic reaction (blue sphere); then it can bind the substrate of the exergonic reaction (green square). Together, these binding reactions drive the system’s conformational change (yellow to orange). In this state, the system is able to complete the endergonic reaction (sphere turning from blue to purple) and allow the release of that product. Afterward, the system completes the exergonic reaction (green square to red square) through product release. This system therefore features coupling in which the endergonic reaction is completed before the exergonic one, provided that the reagents of both reactions are binding at the same time before release of the first product.

Figure 1 illustrates the operational scheme of the coupling complex proposed by Branscomb et al., which was designed to achieve endergonic synthesis [19]. In this reaction network, the reagent for the driven reaction (endergonic) binds first to the coupling molecule, followed by the reagent for the driving reaction (exergonic). The complex then undergoes a conformational change, enabling the formation and release of the driven reaction product, followed by the exergonic product. Critically, the complex returns to its initial state only upon the completion of both reactions, reflecting a double-gating mechanism [20]. This ensures that the less probable product of the driven reaction is synthesized before the more likely product of the driving reaction, adhering to the logic: “neither reaction completes unless both do.” Such mechanisms exemplify how precise coupling enables the molecular-scale realization of intricate energy and material transformations.

The scenario depicted in Figure 1 assumes a design where the product of the endergonic reaction is generated and released before the product of the exergonic reaction. However, alternative designs are theoretically possible and may offer additional insights into the dynamics of coupling systems [19]. In addition, the reaction network proposed by Branscomb et al. does not explicitly take into account all the other reactions that in principle could take place among the different molecules [19]. Finally, a quantitative analysis of rates and yields for such a network was never carried out. To fill these relevant gaps, in our computational study, various reaction pathways were systematically explored to evaluate qualitatively and quantitatively the feasibility and efficiency of different coupling configurations. In particular, to investigate the relationship between network structure and behavior, we carried out a thorough computational kinetic analysis, incorporating the two coupled reactions with appropriately assigned Gibbs free energy values. Variations in these energy parameters allowed for the generation of distinct models, which were rigorously tested to simulate various coupling scenarios. Through these simulations, the fundamental requirements for effective coupling were identified, including the roles of activation energy barriers and energy landscapes in governing the reaction pathways. Additionally, parameter adjustments were performed to probe the limits of the models and explore strategies for optimizing their efficiency, providing valuable insights into the design principles of functional and robust coupling systems.

It turns out that the principle of double-gating remains critical across all designs: the exergonic reaction cannot proceed to completion unless the endergonic reaction also reaches completion, and vice versa [20]. This mutual dependency ensures that the formation of the thermodynamically unfavorable product of the endergonic reaction kinetically facilitates the completion of the exergonic reaction, thereby tightly coupling the two processes.

## 2. Results

### 2.1. The Reaction Network

The exergonic reaction is defined as:

(1) $$A+X\;\overset{M}{⇌}\;B+Y$$

where A and X are the reagents of the exergonic reaction and B and Y are its products. The endergonic reaction is defined as:

(2) $$C+W\;\overset{M}{⇌}\;D+Z$$

C and W are the reagents of the endergonic reaction and D and Z are its products. The bi-products Y and Z are considered waste while B and D are the wanted products. The exergonic and endergonic reactions are chemically independent from each other, sharing only the same coupling complex, M, which can also be considered a catalyst. The initial concentrations of the species in this network are collected in Table 1, along with the intermediates involved in the possible reaction pathways.

**Figure 2:**
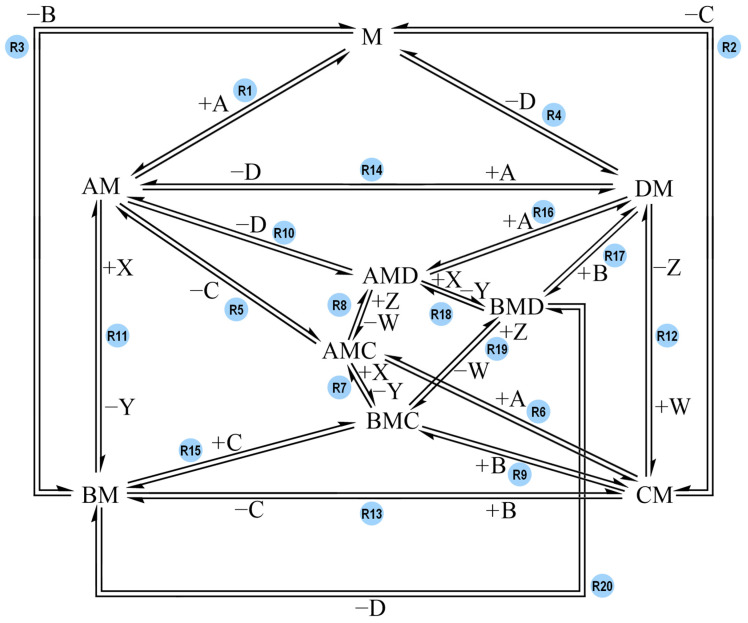
The general reaction network that was designed to study coupling between the exergonic reaction and the endergonic one. Each reaction is labelled with its own reaction name (e.g., R1). A and X are the reagents of the overall exergonic reaction, and B and Y are its products. C and W are the reagent and product of the overall endergonic reaction, and D and Z are its products. B and D are the products of interest whereas Y and Z are treated as waste. M acts as the complex coupling the reactions. All possible reaction paths for an overall A-to-B (exergonic) and C-to-D (endergonic) conversion are featured. For the sake of readability, only one direction of each reaction is labelled with reagents binding to M and/or products being released from the complex (e.g., +A). It is implied that the reaction in the opposite direction has opposite signs for said species.

The network that connects all these species links the species together through all the possible reactions. This network features twenty reaction steps, presented in Figure 2. For the sake of readability, only one direction of each reaction is labelled with reagents binding to M and/or products released from the complex (e.g., +A). It is implied that the reaction in the opposite direction has opposite signs for said species.

The network represents all the potential paths that can lead to B and D synthesis with a coupling complex, M. The model is meant to be as inclusive as possible for a hypothetical coupling system and should help evaluate the effects of the different pathways on the performance for effective coupling.

Arbitrary G° values (ranging between 0 and 21 kcal mol^−1^) were assigned to the species listed in Table 1 to create various models (which will be discussed in the following sections): Model-0, Model-1, Model-2, and Model-3. For each model, the temperature was set to 298 K, the reaction steps’ ΔG° values were calculated and the values of their activation barriers (forward and backward) were arbitrarily assigned. According to the reaction scheme proposed by Branscomb et al. (Figure 1), the endergonic reaction takes place before the exergonic one: the reagent of the driven reaction binds to the complex before the reagent of the driving reaction, and the product of the driven reaction is generated and released before the driving one [19]. The reaction pathway proposed in Figure 1 corresponds to the following reaction sequence in our network (Figure 2): R2 + R6 + R8 + R10 + R11 + R3 (in this direction: C + M → CM, CM + A → AMC, AMC + W → AMD + Z, AMD → AM + D, AM + X → BM + Y, BM → B + M). According to this reaction pathway, C binding to M is followed by A binding to CM, and D is generated and released from the complex before B is generated and released as well. The models tested in COPASI were designed in order to check if that order of reaction steps is the one and only pathway that allows coupling, or if there are other reaction sequences that successfully lead to coupling. The concentration of species A, B, C and D were plotted against time to see if the models could couple the reactions together and generate the desired products, B and D.

### 2.2. Model-0

**Figure 3:**
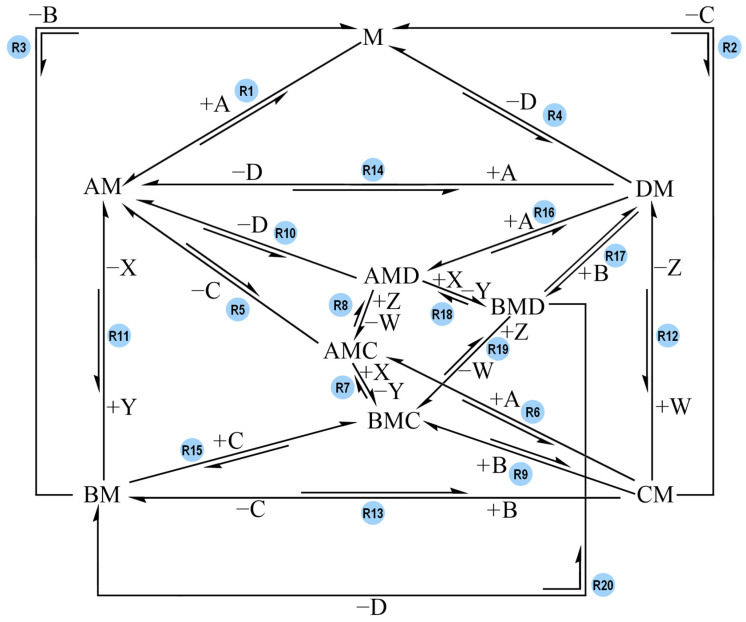
Reaction network of Model-0 and Model-0-B. Each reaction is labelled with its own reaction name (e.g., R1). A and X are the reagents of the overall exergonic reaction, and B and Y are its products. C and W are the reagent and product of the overall endergonic reaction, and D and Z are its products. B and D are the products of interest whereas Y and Z are treated as waste. M acts as the complex coupling the reactions together. All possible reaction paths for an overall A-to-B (exergonic) and C-to-D (endergonic) conversion are featured. For the sake of readability, only one direction of each reaction is labelled with reagents binding to M and/or products being released from the complex (e.g., +A). It is implied that the reaction in the opposite direction has opposite signs for said species.

Starting with Model-0, the ΔG° values of each reaction were calculated under the condition that the overall ΔG°_A-to-B_ was equal to −5 kcal mol^−1^ regardless of which reaction pathway would generate B, and the overall ΔG°_C-to-D_ was equal to +2 kcal mol^−1^, regardless of which reaction pathway would generate D. The G° values assigned to each reaction species were arbitrarily chosen (Table 2), under the constraints that ΔG°_A-to-B_ = −5 kcal mol^−1^ and ΔG°_C-to-D_ = +2 kcal mol^−1^. Figure 3 shows the reaction network of Model-0.

The ΔG° values of the individual pathways were calculated by assigning the G° values reported in Table 2. For Model-0, the reactions, their ΔG° values, their activation barriers and their rate constants are reported in Table 3. The activation barriers, E_a(forward)_ and E_a(backward)_, were arbitrarily selected under the constraint imposed by the ΔG° values (i.e., E_a(forward)_ = E_a(backward)_ + ΔG°), and the activation barriers were in turn used to calculate the rate constants with Equation (3). The species and their concentrations and the reactions of the network and their rate constants were used in COPASI in order to run simulations and obtain a plot of concentration vs. time.

**Figure 4:**
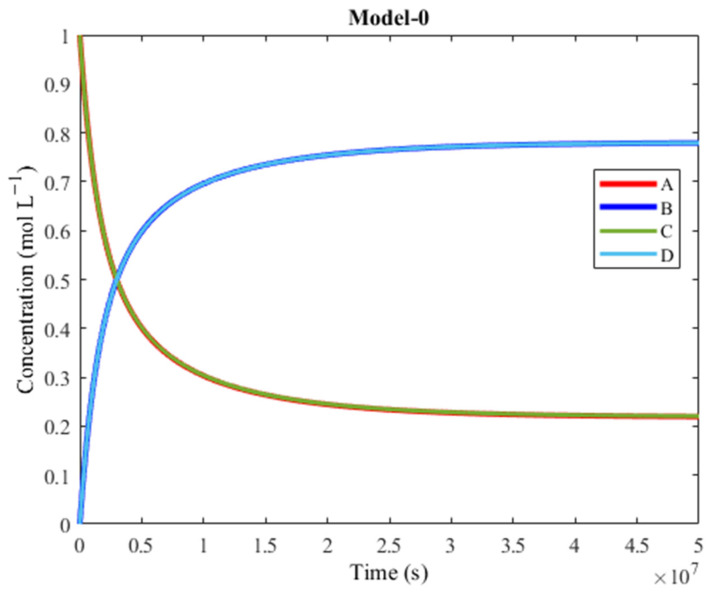
Plot of concentration vs. time of Model-0. A is converted to B (exergonic) and C is converted to D (endergonic), thanks to coupling. There is great overlap between the concentrations of the two products and of the two reagents, respectively. The products’ final yield is over 70%, which makes this model efficient, but its timescale makes it extremely slow.

The resulting plot is presented in Figure 4. The reagents X and W are omitted (they overlap with the A and C curvature, so their inclusion would be unnecessary). The bi-products Y and Z are also omitted because they are considered waste (but they do overlap with the B and D curvature).

The plot of Model-0 shows that the concentration of D increases above the concentration of C, despite D being the unfavorable product of the endergonic reaction. Considering the general overlap between the concentrations of B and D, and the concentrations of A and C respectively, the products are generated approximately at the same rate and the reagents are consumed approximately at the same rate due to coupling. The products’ final yield is beyond 70%. Model-0 represents a network in which M acts as a complex that couples A + X $\overset{M}{⇌}$ B + Y and C + W $\overset{M}{⇌}$ D + Z. The time it takes for the synthesis to complete is over 10^7^ s, meaning that the model is very slow.

The R2 + R6 + R8 + R10 + R11 + R3 pathway proposed in Figure 1 (C + M → CM, CM + A → AMC, AMC + W → AMD + Z, AMD → AM + D, AM + X → BM + Y, BM → B + M) still works while omitting all other pathways, but at a much higher timescale (10^16^ s instead of 10^7^ s). In order to improve the timescale of the R2 + R6 + R8 + R10 + R11 pathway, the activation barriers of R10 and R11 were lowered (Table 4).

**Figure 5:**
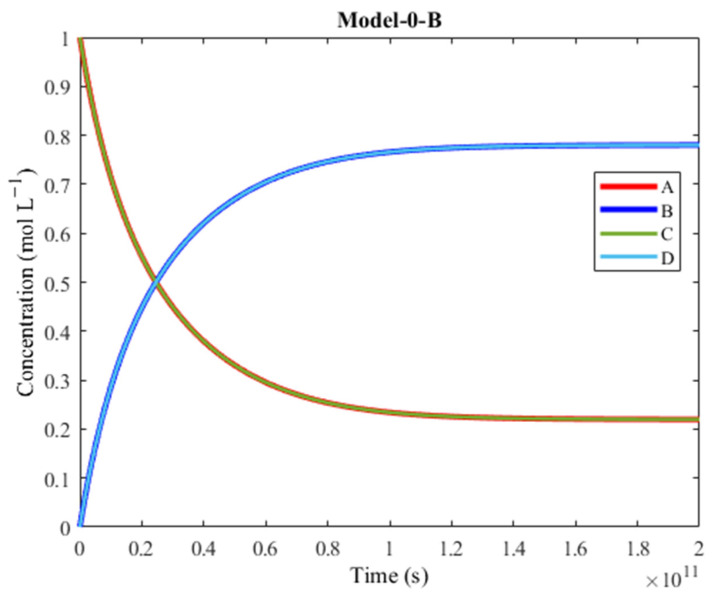
Plot of concentration vs. time of Model-0-B. A is converted to B (exergonic) and C is converted to D (endergonic), thanks to coupling. Model-0-B exclusively contains the R2 + R6 + R8 + R10 + R11 + R3 pathway, whereas Model-0 also includes other pathway possibilities featured in Figure 3.

This led to Model-0-B (Figure 5), which features only the reaction steps presented in Table 4.

This model is still very slow (10^11^ s timescale). The exceptionally slow timescale can be attributed to the unfavorable directionalities of reactions R2, R8 and R11. Focusing on the R2 + R6 + R8 + R10 + R11 + R3 pathway, a new model was designed by assigning new G° values to the network’s species in order to obtain negative ΔG° values for all six reaction steps in the direction of interest: C + M → CM, CM + A → AMC, AMC + W → AMD + Z, AMD → AM + D, AM + X → BM + Y, BM → B + M. This model was called Model-1.

### 2.3. Model-1

Model-1 was created by assigning new random G° values (ranging between 0 and 21 kcal mol^−1^), such that all six reactions of the R2 + R6 + R8 + R10 + R11 + R3 pathway would be spontaneous in the direction that leads to the wanted products. These G° values (Table 5) still ensured that the overall exergonic reaction was spontaneous and the overall endergonic reaction was still non-spontaneous: ΔG°_A-to-B_ = −15 kcal mol^−1^ and ΔG°_C-to-D_ = +2 kcal mol^−1^.

**Figure 6:**
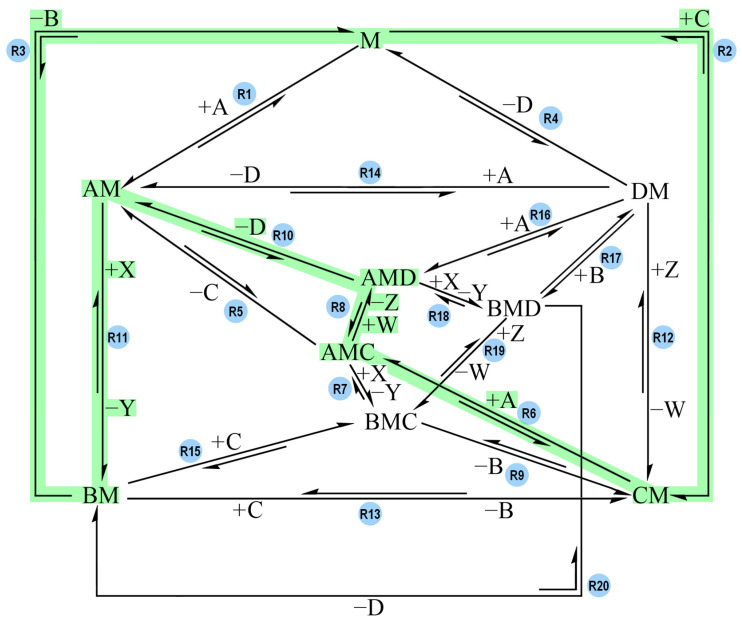
Reaction network of Model-1. The pathway highlighted in green (R2 + R6 + R8 + R10 + R11 + R3) is called Track 1 and it represents the following sequence in the direction of product synthesis: C + M → CM, CM + A → AMC, AMC + W → AMD + Z, AMD → AM + D, AM + X → BM + Y, BM → B + M. Each reaction is labelled with its own reaction name (e.g., R1). A and X are the reagents of the overall exergonic reaction, B and Y are its products. C and W are the reagent and product of the overall endergonic reaction, D and Z are its products. B and D are the products of interest whereas Y and Z are treated as waste. M acts as the complex coupling the reactions together. All possible reaction paths for an overall A-to-B (exergonic) and C-to-D (endergonic) conversion are featured. For the sake of readability, only one direction of each reaction is labelled with reagents binding to M and/or products being released from the complex (e.g., +A). It is implied that the reaction in the opposite direction has opposite signs for said species.

The network of Model-1 is shown in Figure 6, with the R2 + R6 + R8 + R10 + R11 + R3 pathway being highlighted in green. This pathway was named Track 1.

In order to favor the six reaction steps of Track 1, the other pathways were essentially shut down by assigning high activation barriers to all the other individual reactions featured in the network. The values of Model-1 are listed in Table 6.

**Figure 7:**
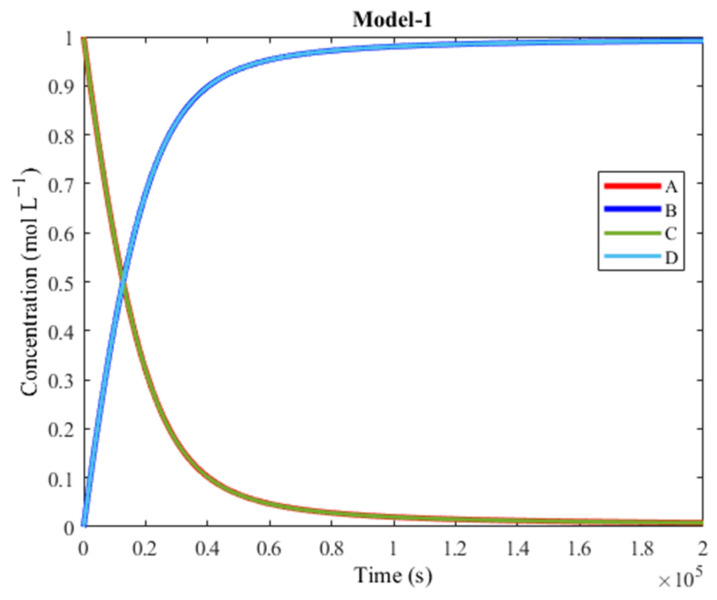
Plot of concentration vs. time of Model-1. A is converted to B (exergonic) and C is converted to D (endergonic), thanks to coupling. There is great overlap between the concentrations of the two products and of the two reagents, respectively. The final concentration of the products is 100%, a complete conversion.

Figure 7 shows the plot of Model-1, which is at a 10^5^ s timescale.

The system reaches constant concentrations at around 2 × 10^5^ s, which is approximately two days and seven hours. The plot shows overlap between products, proving that coupling is successful. In terms of timescale, Model-1 is 250 times faster than Model-0. Moreover, Model-1 provides a 100% yield and excellent overlap between the products. This model also demonstrates that the correct and most efficient pathway for coupling depends on the spontaneity of the individual reaction steps (in this case, Track 1). In order to ensure that coupling could be attributed to Track 1, all the other reactions were omitted from the network and a simulation was run with just the six reaction steps of Track 1. The resulting plot was identical to the one shown in Figure 7, thus proving that this pathway was indeed the coupling one, while the other pathways were hindered by high activation barriers. As long as the overall endergonic reaction remains non-spontaneous, the most efficient coupling pathway is the one featuring reaction steps that are all spontaneous toward the direction that leads to B and D synthesis.

### 2.4. Model-2

Theoretically, it should be possible to achieve coupling with other reaction sequences besides Track 1 (C + M → CM, CM + A → AMC, AMC + W → AMD + Z, AMD → AM + D, AM + X → BM + Y, BM → B + M). By lowering the activation barriers of other reaction steps, Model-1 was repurposed to test other reaction pathways within the network as shown in Figure 6. The purpose of these simulations was to see if other pathways would allow coupling or interfere with Track 1. Table 7 lists the reaction sequences that were tested, one at a time, in COPASI. All these tracks retained the G° values listed in Table 5 and the directionalities seen in Model-1.

**Figure 8:**
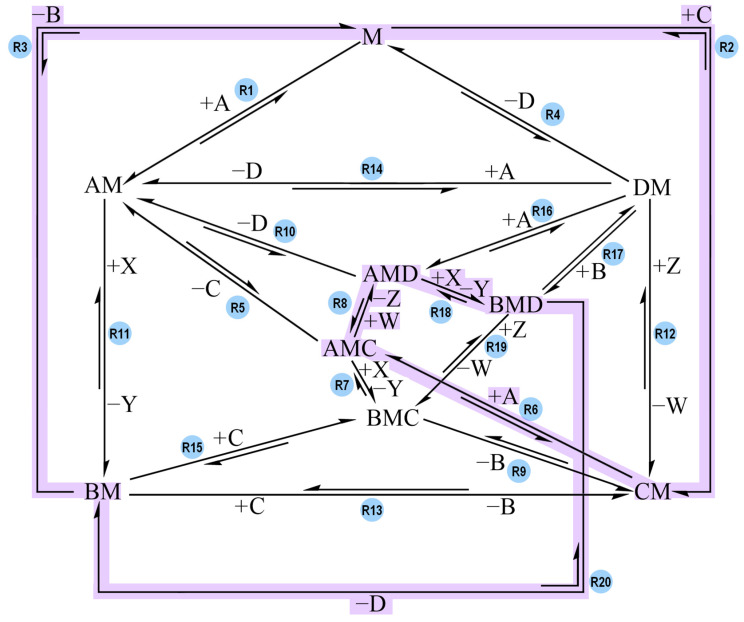
Reaction network with the same directionalities as the ones in Figure 6. The R2 + R6 + R8 + R18 + R20 + R3 pathway is highlighted in purple and is called Track 2. Track 2 represents the following sequence in the direction of product synthesis: C + M → CM, CM + A → AMC, AMC + W → AMD + Z, AMD + X → BMD + Y, BMD → BM + D, BM → B + M.

Each reaction step of each track had its activation barriers lowered (see Supplementary Materials for the tables and plots of each track). The reaction sequences were first tested by still letting Track 1 have the same values as in Model-1. The plots showed how the new tracks affected Track 1 and whether or not coupling was still achievable. For example, Track 1 and Track 2 were allowed to coexist with low activation barriers. The presence of Track 2 did not interfere with the behavior that was observed when only considering Track 1, whereas other reaction sequences prevented coupling, leading to time course plots with no overlap and low yield for the endergonic reaction. Then, new simulations were run with the new tracks alone: only one track had low activation barriers assigned to it, while all the other reactions in the network were omitted, including the six reactions of Track 1. Figure 8 highlights Track 2 in purple (this network’s directionalities are identical to the one in Figure 6).

**Figure 9:**
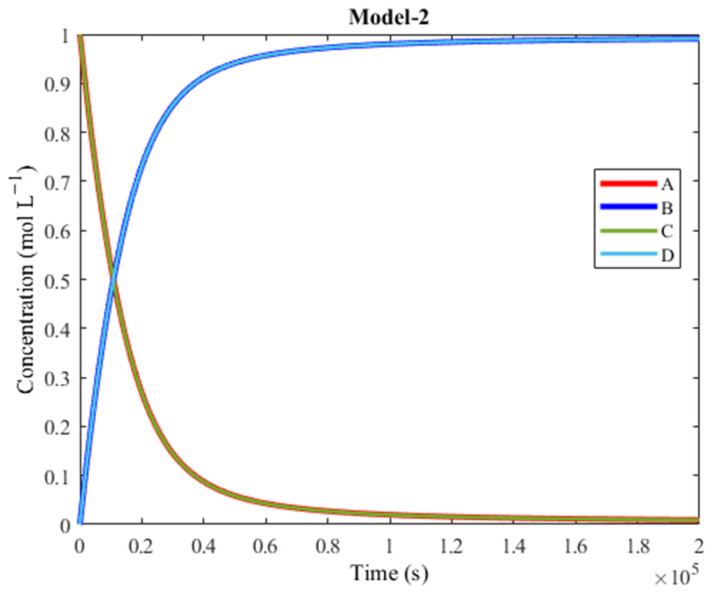
Plot of concentration vs. time of Model-2. A is converted to B (exergonic) and C is converted to D (endergonic), thanks to coupling. This plot shows that the performance of Model-2 is on par with Model-1. Model-2 relies on Track 2, a different pathway compared to Model-1, whose performance depends on Track 1.

For the six reactions involved in Track 2, the activation barriers were lowered, while all the other reactions within the network were given high activation barriers. The reactions with high barriers (the ones that are not highlighted in purple in Table 8) essentially became inaccessible (deleting such reaction steps does not affect the plot), ensuring that the network would rely only on Track 2. A Track-2-only system was thus obtained, and it was called Model-2. Table 8 lists the values of Track 2 and Figure 9 shows the plot of Model-2.

The plot in Figure 9 shows that the performance of Model-2 is comparable to Model-1. The plot of Model-2 is very similar to Model-1, but the intersection point in Model-2 is reached sooner than in Model-1 (10,816 s instead of 12,745 s). The plot of Model-2 demonstrated that Track 2 was able to achieve coupling on its own, even when Track 1 was no longer available within the reaction network. Track 2 was able to achieve coupling on its own because, much like Track 1, its reaction steps are all spontaneous in the direction that leads to product synthesis.

Track 3, Track 4, Track 5, Track 6 and Track 7 worsen the performance of Track 1. With the exception of Track 7, all the reaction sequences interfere with Track 1 because their simultaneous presence with Track 1 creates a “short circuit” that allows the exergonic reaction to occur without coupling: R1 + R11 + R3 (A + M → AM → BM → B + M).

**Figure 10:**
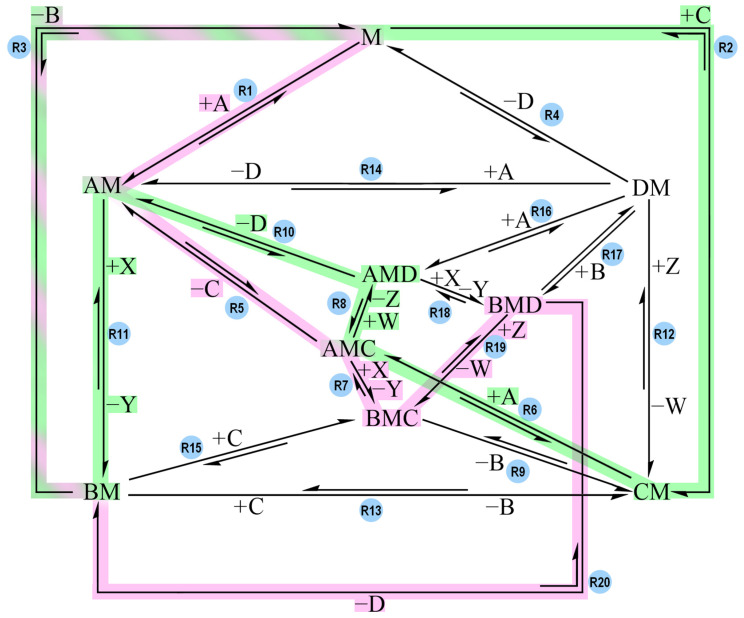
Reaction network with the same directionalities as the ones in Figure 6. The pathway highlighted in green (R2 + R6 + R8 + R10 + R11 + R3) is called Track 1. The pathway highlighted in pink (R1 + R5 + R7 + R19 + R20 + R3) is called Track 4. Track 1 and Track 4 share the final reaction step: R3. When only one of the two tracks has low activation barriers, coupling is observable (Track 4 shows coupling, but at a slower timescale than Track 1). However, if Track 1 and Track 4 both have low activation barriers, then coupling is lost because their combination allows the exergonic reaction to complete independently via R1 + R11 + R3.

For example, if Track 4 (R1 + R5 + R7 + R19 + R20 + R3) and Track 1 are the only pathways with low activation barriers while the others have high barriers, then the combination of their reaction steps can cause the exergonic reaction to occur independently from the endergonic one (Figure 10).

**Figure 11:**
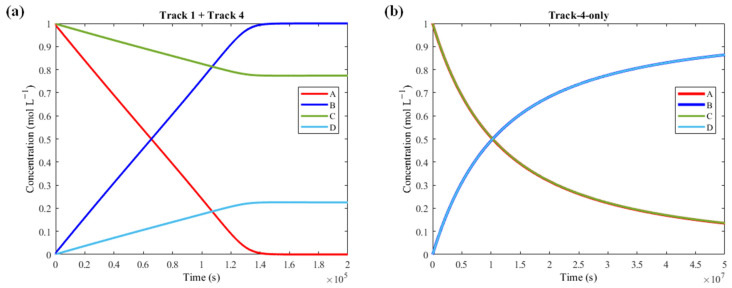
(**a**) Plot obtained by keeping all activation barriers high, except for Track 1 (R2 + R6 + R8 + R10 + R11 + R3) and Track 4 (R1 + R5 + R7 + R19 + R20 + R3), whose reaction steps have low activation barriers. (**b**) Plot obtained by keeping all activation barriers high, except for Track 4, whose reaction steps have low activation barriers. Overlap is observable. Coupling is therefore computationally possible with just Track 4, but it is very slow.

A is able to bind to M thanks to R1 (made accessible thanks to Track 4), then AM converts to BM through R11 (made accessible thanks to Track 1) and B is released via R3 (the final step of both tracks). Thus, when Track 1 and Track 4 are available simultaneously, the R1 + R11 + R3 pathway becomes accessible, and coupling is lost (Figure 11a, see Supplementary Materials for the tables). However, if Track 4 is the only available reaction sequence within the network while Track 1 has high activation barriers, then coupling is observable due to the directionalities of its reaction steps (Figure 11b).

**Figure 12:**
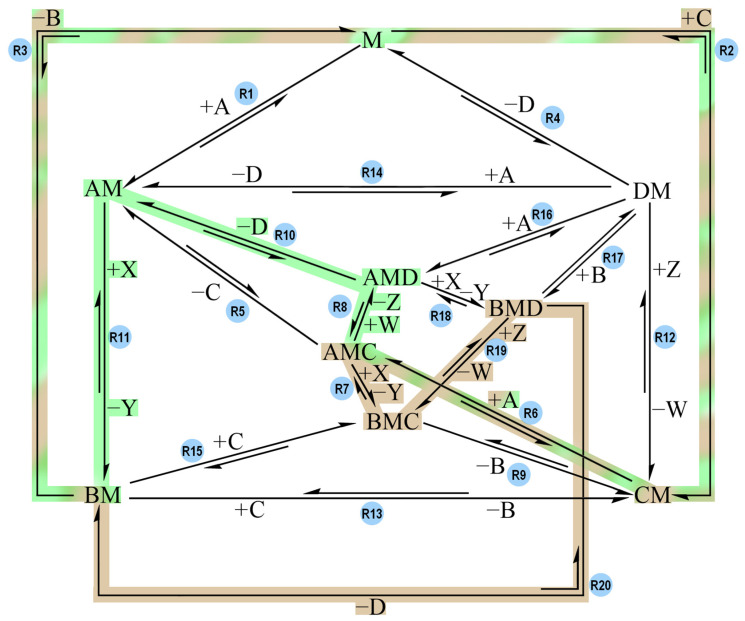
Reaction network with the same directionalities as the ones in Figure 6. The pathway highlighted in green (R2 + R6 + R8 + R10 + R11 + R3) is called Track 1. The pathway highlighted in brown (R2 + R6 + R7 + R19 + R20 + R3) is called Track 7. Track 1 and Track 7 share the three reaction steps R2, R6 and R3. When Track 1 and Track 7 both have low activation barriers, coupling is observable, but Track 7 severely slows down the process. Track 7 alone shows coupling, but at a slower timescale than Track 1.

The same reasoning applies to Track 3, Track 5, Track 6, and Track 7. The combination of Track 1 and Track 7 does not create the “short circuit” scenario (Figure 12, see Supplementary Material for the tables and plots).

Track 7 hinders the performance of Track 1 because the intermediate BMC competes with AMD: AMC is converted to AMD via R8, following the Track 1 sequence (like in Model-1). However, since Track 7 is also available, AMC can become BMC via R7 instead of turning into AMD. The intermediate BMC has to either turn back into AMC and then proceed via Track 1, or it has to deal with the unfavorable directionality of R19 in order to keep going via Track 7. Overall, Track 7 (R2 + R6 + R7 + R19 + R20 + R3) is able to achieve coupling, but at a slower timescale compared to what was seen in Model-1 (2 × 10^7^ s as opposed to 2 × 10^5^ s), due to the unfavorable directionality of R19.

Considering Model-2, it can be deduced that any reaction sequence could potentially lead to coupling as fast as Model-1, provided that each reaction step has the right directionality and low activation barriers. One final model (Model-3) was thus designed to solidify the notion that coupling is possible regardless of the order of reaction steps while achieving coupling at a timescale similar to Model-1 and Model-2. Coupling should be possible regardless of which reagent binds first or which product is generated and released first.

### 2.5. Model-3

Model-3 was inspired by Track 3 (R1 + R5 + R7 + R19 + R17 + R4). Track 3 is essentially the antithesis of Track 1. In Track 1, C binds to M before A and D is generated and released before B. In Track 3, A binds to M before C does, and B is generated and released before D. Track 3 is the following sequence: A + M → AM, AM + C → AMC, AMC + X → BMC + Y, BMC + W → BMD + Z, BMD → DM + B, DM → D + M.

New random G° values were assigned with the constraint that all six reactions of Track 3 would be spontaneous in the direction that leads to the wanted products. These G° values (Table 9) still ensured that the overall exergonic reaction was spontaneous and the overall endergonic reaction was still non-spontaneous, with ΔG°_A-to-B_ = −19 kcal mol^−1^ and ΔG°_C-to-D_ = +2 kcal mol^−1^.

**Figure 13:**
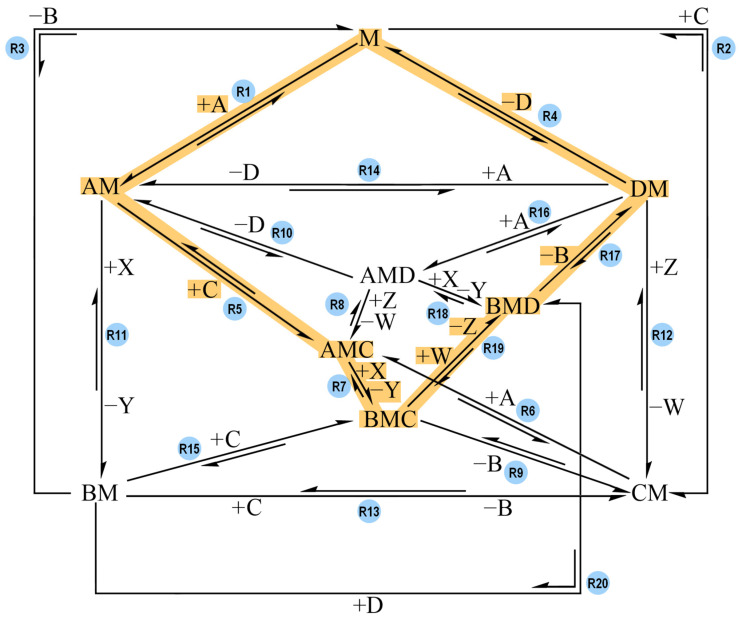
Reaction network of Model-3. The pathway highlighted in orange (R1 + R5 + R7 + R19 + R17 + R4) is called Track 3. In this model, Track 3 features all directionalities in favor of product synthesis.

Figure 13 shows the network of Model-3, with Track 3 highlighted in orange.

The activation barriers of the reaction steps of Track 3 were lowered while the rest were given high values, such that Track 3 was the only coupling pathway accessible within the network (Table 10).

**Figure 14:**
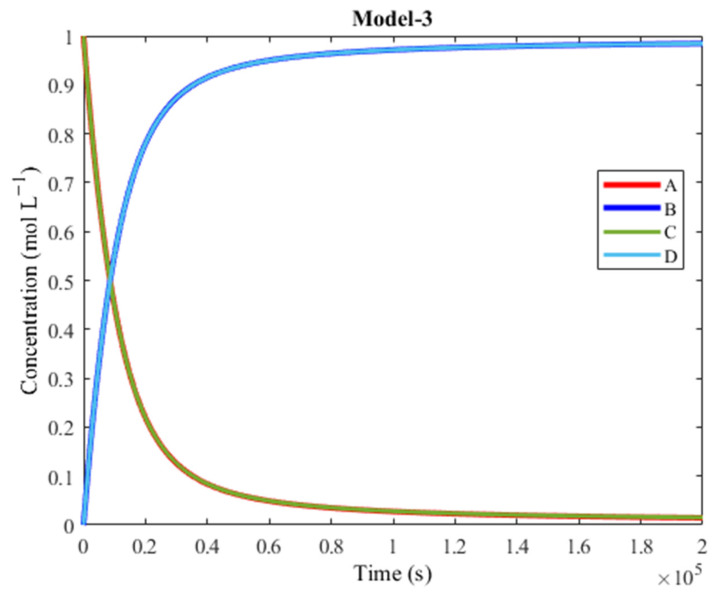
Plot of concentration vs. time of Model-3. A is converted to B (exergonic) and C is converted to D (endergonic), thanks to coupling.

As seen in Figure 14, the plot of Model-3 is similar to those of Model-1 and Model-2, featuring an overlap between the products’ concentrations and an overlap between the reagents’ concentrations.

The plot of Model-3 reaches the intersection point quicker than in Model-1 and Model-2, but it takes longer to reach 100% yield, at approximately 1.3 × 10^6^ s (15 days). Still, more than 90% of the products are synthesized within 2 × 10^5^ s, so its performance is still comparable to Model-1 and Model-2.

## 3. Discussion

Considering the obtained time course plots, Model-1 and Model-2 proved that there are two routes that achieve coupling within a 2 × 10^5^ s. One is the reaction sequence proposed by Branscomb et al., which corresponds to Track 1 [19]. The other pathway that performs just as well and just as fast is Track 2, seen in Model-2. This pathway shares most of its reaction steps with the former, but B is generated before D unbinds from M (B and D are binding simultaneously in the intermediate BMD).

Model-3 is slower at achieving 100% yield compared to Model-1 and Model-2. However, Model-3 reaches 50% yield sooner than the other models. The intersection point being reached sooner in Model-3 than in previous models can be attributed to the different activation barriers involved. Specifically, in Model-3, the forward activation barrier of R1 is very low (0.1 kcal mol^−1^), while its backward activation barrier is relatively high (9.1 kcal mol^−1^). A is therefore much more likely to bind to M (forward process) than it is to leave it (backward process), which is very beneficial for the forward directionality in Model-3. The average difference between backward and forward barriers of Track 3 in Model-3 is higher (2.8 kcal mol^−1^) than the average difference of the backward and forward barriers of Track 1 in Model-1 and Track 2 in Model-2 (2.2 kcal mol^−1^ for both). This suggests that Model-3 is less likely to reverse backward, on average.

The reaction sequences listed in Table 7 showed how the various pathways within the same network could influence one another. Many tracks combined with Track 1 hindered coupling by allowing the exergonic reaction to complete on its own via the R1 + R11 + R3 pathway (A + M → AM → BM → B + M), which does not involve the endergonic reaction (as seen in Figure 10 and Figure 11a). The exergonic reaction is able to complete on its own thanks to the low activation barriers and favorable directionalities of R1, R3 and R11. This scenario can be referred to as a “short circuit”.

Different reaction sequences achieved coupling with a combination of favorable and unfavorable directionalities, but the unfavorable directionalities led to very long timescales. This was the case for the reaction sequences listed in Table 7. In retrospect, Model-1 is slow for the same reason: some of its reaction steps are spontaneous in the opposite direction with respect to the desired directionality. The network of Model-1 features multiple pathways that may result in coupling, but, together, they slow down the overall process due to combinational effects.

**Figure 15:**
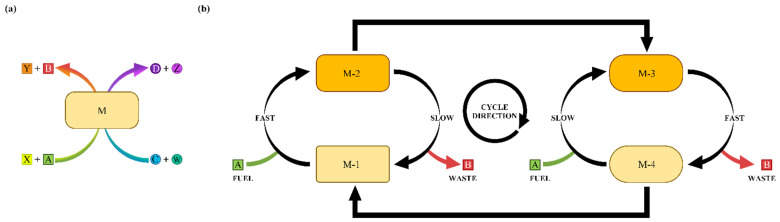
(**a**) Schematic representation of the Janusian enzyme, called M. A and X are the reagents of the exergonic reaction and B and Y are its products. C and W are the reagents of the endergonic reaction and D and Z are its products. M changes state when the other species are binding, allowing coupling to occur, resulting in the release of both B and D. The various states of M are omitted here because this scheme would otherwise become as intricate as the network in Figure 2. (**b**) Schematic representation of an information ratchet mechanism, like those studied by Leigh and collaborators [20], featuring A-to-B as the fuel-to-waste reaction. The ratchet changes states (M-1, M-2, M-3 and M-4) thanks to the following directional path: M-1 is converted to M-2, then it becomes M-3, which then relaxes to M-4.

The species M described in the models acts as a coupling enzyme, which was defined as a “Janusian enzyme” due to its ability to catalyze the endergonic reaction as well as the exergonic one [19]. The Janusian enzyme acts as a mediator for the coupling of the A-to-B and C-to-D reactions. The enzyme M changes state depending on the various available pathways (e.g., M becomes BMD), and is able to catalyze the formation of the endergonic product D, along with product B. In contrast, in the case of most of the synthetic molecular ratchets experimentally investigated in the literature, such as the rotaxanes finely designed in the laboratories of Leigh and collaborators [20,21], the catalyst itself undergoes an endergonic transformation, featuring a fuel-to-waste process as the exergonic reaction. The difference between the Janusian enzyme initially proposed by Branscomb et al. and an information ratchet such as the finely designed rotaxanes by Leigh and collaborators are illustrated in Figure 15 [19,20].

The scheme presented in Figure 15a is a simplified version of the network shown in Figure 2, omitting the various states of the enzyme by simply representing it as M, with A and C binding and turning into B and D in parallel. In contrast, Figure 15b shows an information ratchet that is composed of only two key species, A and M, like those designed by Leigh and collaborators [20,21]. Therefore, the Janusian enzyme takes endergonic synthesis a step forward by acting as a mediator rather than a final product, allowing the synthesis of a hypothetical endergonic reaction (C-to-D) by coupling it with an exergonic one (A-to-B). However, the simulations of Model-0 and Model-0-B showed that this coupling method can be very slow. The successful generation of product B and D and their overlapping high-percentage concentrations come with the price of a very long time scale. Indeed, the high efficiency that was achieved in Model-0 was at the expense of the time it took to complete the synthesis. Model-1 and Model-2 achieved coupling with 100% yield in 2 × 10^5^ s. While this duration is plausible for artificial endergonic synthesis, such a timescale is still not comparable to biological enzymes that are vital for life. These observations are relevant not only because endergonic synthesis in biology requires faster time scales compared to the time scales of the reported models, but also because endergonic synthesis by means of information ratcheting systems is expected to be relevant in industrial processes [22,23,24].

## 4. Materials and Methods

COPASI 4.39 was used as software to carry out all simulations of the models presented in the next section. COPASI is an open-source software application for simulation and analysis of biochemical networks and their dynamics. The COPASI project is an international collaboration between three groups at the Biocomplexity Institute and Initiative at University of Virginia, the University of Heidelberg, and the University of Connecticut, UConn Health [25].

For each model, the rate constants of the forward and backward reactions were calculated using the Arrhenius equation, keeping the pre-exponential factor equal to 1:

(3) $$k=e^{\left(\frac{-E_{a}}{RT}\right)}$$

E_a_ is the activation energy of a reaction expressed in kcal mol^−1^, R is the universal gas constant expressed as 1.987 × 10^−3^ kcal K^−1^ mol^−1^, and the temperature T is expressed in K. The calculated rate constants were set up in COPASI to study each model’s behavior. Then, using COPASI’s time course feature, the concentrations of the reagents and products of the coupled reactions were plotted against time to see if the system could couple the reactions and generate the desired products.

## 5. Conclusions

Several hypothetical reaction networks that couple endergonic synthesis with orthogonal exergonic processes were designed and investigated by computational simulations.

The models were put to the test in COPASI to study scenarios differing for free and activation energies. The models’ overall network included the sequence of reactions proposed by Branscomb et al., as well as other pathway options with different directionalities, proving that other designs are possible in principle. In Model-0, the products’ yield was over 70% on a 5 × 10^7^ s timescale. Model-1, Model-2 and Model-3 were much faster than Model-0 and reached 100% yield. The different models computationally proved that coupling still works regardless of which reagent binds first or which product is generated and released first. Our investigation demonstrated how to study the behavior of such reaction networks: possible collateral reactions must be taken into account, as they can impact the system’s performance and even prevent coupling (e.g., the exergonic reaction completes by itself when Track 1 and Track 4 have low activation barriers at the same time).

Despite the complexity of the overall network, combinational effects were rationalized for several scenarios that featured one or two reaction sequences at the same time. The general condition for efficient and fast coupling was assigning desirable directionality to all the reaction steps of a specific pathway. Future works may consider the development of a dedicated computational protocol that can automatically set up the various combinations of the parameters of a given network, thus allowing a more systematic exploration of hypothetical models in more depth and in more variety. Such an approach would allow for determining if it is possible to combine thermodynamics and kinetic parameters that, while remaining consistent with microscopic reversibility, might allow high yields and fast overall reaction rates, or if a tradeoff between yield and rates is unavoidable. What would be most interesting is turning this specific computational analysis into a generally applicable method for predicting the reactivity of coupling complexes. On a less theoretical note, future collaborations in the field of synthetic chemistry could lead to the design and exploration of actual coupling complexes that can perform like a Janusian enzyme and potentially open a path to new ways of endergonic synthesis.

## Figures and Tables

**Table 1:** Initial concentrations of all species featured in all the models for the coupling of two exchange reactions. A and X are the reagents of the overall exergonic reaction, and B and Y are its products. C and W are the reagent and product of the overall endergonic reaction, and D and Z are its products. B and D are the products of interest whereas Y and Z are treated as waste. M acts as the complex coupling the reactions together. The rest of the species are intermediates made by binding reagents/products to M (e.g., AM is the intermediate featuring A binding to M).

Species	Initial Concentration (mol L^−1^)
A	1
B	0
C	1
D	0
X	1
Y	0
W	1
Z	0
M	0.001
AM	0
BM	0
CM	0
DM	0
AMC	0
BMC	0
AMD	0
BMD	0

**Table 2:** Assigned standard Gibbs free energies of the species featured in Model-0. These values are arbitrarily chosen under the condition that ΔG°_A-to-B_ = −5 kcal mol^−1^ and the ΔG°_C-to-D_ = +2 kcal mol^−1^ regardless of which intermediates are used to achieve A-to-B and C-to-D conversions.

Species	G° (kcal mol^−1^)
A	10
B	5
C	5
D	7
M	0
X	0
Y	0
W	0
Z	0
AM	8
BM	9
CM	13
DM	12
AMC	15
BMC	13
AMD	18
BMD	17

**Table 3:** List of all the reactions featured in Model-0. Each reaction has a label in line with the diagram of Figure 3. All the reactions are listed in the direction of spontaneity from right to left (e.g., A + M ⇌ AM means conversion from A to AM is spontaneous, so its standard Gibbs free energy change, ΔG°, is negative). The forward and backward activation energy barriers are listed as E_a(forward)_ and E_a(backward)_. The forward and backward reaction rates (k, k_−1_) are calculated using Equation (3).

ID	Reaction	ΔG° (kcal mol^−1^)	E_a(forward)_ (kcal mol^−1^)	E_a(backward)_ (kcal mol^−1^)	k	k_−1_
R1	A + M ⇌ AM	−2	4	6	1.17 × 10^−3^	3.98 × 10^−5^
R2	CM ⇌ C + M	−8	1	9	1.85 × 10^−1^	2.51 × 10^−7^
R3	BM ⇌ B + M	−4	1	5	1.85 × 10^−1^	2.15 × 10^−4^
R4	DM ⇌ D + M	−5	2	7	3.41 × 10^−2^	7.35 × 10^−6^
R5	AMC ⇌ AM + C	−2	2	4	3.41 × 10^−2^	1.17 × 10^−3^
R6	CM + A ⇌ AMC	−8	2	10	3.41 × 10^−2^	4.63 × 10^−8^
R7	AMC + X ⇌ BMC + Y	−2	0.1	2.1	8.45 × 10^−1^	2.88 × 10^−2^
R8	AMD + Z ⇌ AMC + W	−3	0.1	3.1	8.45 × 10^−1^	5.33 × 10^−3^
R9	CM + B ⇌ BMC	−5	10	15	4.63 × 10^−8^	9.97 × 10^−12^
R10	AMD ⇌ AM + D	−3	12	15	1.58 × 10^−9^	9.97 × 10^−12^
R11	BM + Y ⇌ AM + X	−1	14	15	5.40 × 10^−11^	9.97 × 10^−12^
R12	CM + W ⇌ DM + Z	−1	14	15	5.40 × 10^−11^	9.97 × 10^−12^
R13	CM + B ⇌ BM + C	−4	11	15	8.56 × 10^−9^	9.97 × 10^−12^
R14	DM + A ⇌ AM + D	−7	8	15	1.36 × 10^−6^	9.97 × 10^−12^
R15	BM + C ⇌ BMC	−1	14	15	5.40 × 10^−11^	9.97 × 10^−12^
R16	DM + A ⇌ AMD	−4	11	15	8.56 × 10^−9^	9.97 × 10^−12^
R17	DM + B ⇌ BMD	0	3	3	6.31 × 10^−3^	6.31 × 10^−3^
R18	AMD + X ⇌ BMD + Y	−1	0.1	1.1	8.45 × 10^−1^	1.56 × 10^−1^
R19	BMD + Z ⇌ BMC + W	−4	0.1	4.1	8.45 × 10^−1^	9.84 × 10^−4^
R20	BMD ⇌ BM + D	−1	0.1	1.1	8.45 × 10^−1^	1.56 × 10^−1^

**Table 4:** List of all the reactions featured in Model-0-B. Each reaction has a label in line with the diagram of Figure 3. All the reactions are listed in the direction of spontaneity from right to left (e.g., CM ⇌ C + M means conversion from CM to C is spontaneous, so its standard Gibbs free energy change, ΔG°, is negative). The forward and backward activation energy barriers are listed as E_a(forward)_ and E_a(backward)_. The forward and backward reaction rates (k, k_−1_) are calculated using Equation (3).

ID	Reaction	ΔG° (kcal mol^−1^)	E_a(forward)_ (kcal mol^−1^)	E_a(backward)_ (kcal mol^−1^)	k	k_−1_
R2	CM ⇌ C + M	−8	1	9	1.85 × 10^−1^	2.51 × 10^−7^
R3	BM ⇌ B + M	−4	1	5	1.85 × 10^−1^	2.15 × 10^−4^
R6	CM + A ⇌ AMC	−8	2	10	3.41 × 10^−2^	4.63 × 10^−8^
R8	AMD + Z ⇌ AMC + W	−3	0.1	3.1	8.45 × 10^−1^	5.33 × 10^−3^
R10	AMD ⇌ AM + D	−3	4	7	1.17 × 10^−3^	7.35 × 10^−6^
R11	BM + Y ⇌ AM + X	−1	1	2	1.85 × 10^−1^	3.41 × 10^−2^

**Table 5:** Assigned standard Gibbs free energies of the species featured in Model-1. These values are arbitrarily chosen under the condition that ΔG°_A-to-B_ = −15 kcal mol^−1^ and the ΔG°_C-to-D_ = +2 kcal mol^−1^ regardless of which intermediates are used to achieve A-to-B and C-to-D conversions.

Species	G° (kcal mol^−1^)
A	20
B	5
C	5
D	7
M	0
X	0
Y	0
W	0
Z	0
AM	11
BM	9
CM	4
DM	12
AMC	21
BMC	13
AMD	19
BMD	17

**Table 6:** List of all the reactions featured in Model-1, with the reaction steps of Track 1 highlighted in green. Each reaction has a label in line with the diagram of Figure 6. All the reactions are listed in the direction of spontaneity from right to left (e.g., A + M ⇌ AM means conversion from A to AM is spontaneous, so its standard Gibbs free energy change, ΔG°, is negative). The forward and backward activation energy barriers are listed as E_a(forward)_ and E_a(backward)_. The forward and backward reaction rates (k, k_−1_) were calculated using Equation (3).

ID	Reaction	ΔG° (kcal mol^−1^)	E_a(forward)_ (kcal mol^−1^)	E_a(backward)_ (kcal mol^−1^)	k	k_−1_
R1	A + M ⇌ AM	−9	21	30	3.97 × 10^−16^	9.94 × 10^−23^
R2	C + M ⇌ CM	−1	0.1	1.1	8.45 × 10^−1^	1.56 × 10^−1^
R3	BM ⇌ B + M	−4	1	5	1.85 × 10^−1^	2.15 × 10^−4^
R4	DM ⇌ D + M	−5	25	30	4.62 × 10^−19^	9.94 × 10^−23^
R5	AMC ⇌ AM + C	−5	25	30	4.62 × 10^−19^	9.94 × 10^−23^
R6	CM + A ⇌ AMC	−3	1	4	1.85 × 10^−1^	1.17 × 10^−3^
R7	AMC + X ⇌ BMC + Y	−8	22	30	7.33 × 10^−17^	9.94 × 10^−23^
R8	AMC + W ⇌ AMD + Z	−2	0.1	2.1	8.45 × 10^−1^	2.88 × 10^−2^
R9	BMC ⇌ CM + B	−4	26	30	8.54 × 10^−20^	9.94 × 10^−23^
R10	AMD ⇌ AM + D	−1	1	2	1.85 × 10^−1^	3.41 × 10^−2^
R11	AM + X ⇌ BM + Y	−2	0.1	2.1	8.45 × 10^−1^	2.88 × 10^−2^
R12	DM + Z ⇌ CM + W	−8	22	30	7.33 × 10^−17^	9.94 × 10^−23^
R13	BM + C ⇌ CM + B	−5	25	30	4.62 × 10^−19^	9.94 × 10^−23^
R14	DM + A ⇌ AM + D	−14	16	30	1.84 × 10^−12^	9.94 × 10^−23^
R15	BM + C ⇌ BMC	−1	29	30	5.38 × 10^−22^	9.94 × 10^−23^
R16	DM + A ⇌ AMD	−13	17	30	3.40 × 10^−13^	9.94 × 10^−23^
R17	DM + B ⇌ BMD	0	30	30	9.94 × 10^−23^	9.94 × 10^−23^
R18	AMD + X ⇌ BMD + Y	−2	28	30	2.91 × 10^−21^	9.94 × 10^−23^
R19	BMD + Z ⇌ BMC + W	−4	26	30	8.54 × 10^−20^	9.94 × 10^−23^
R20	BMD ⇌ BM + D	−1	29	30	5.38 × 10^−22^	9.94 × 10^−23^

**Table 7:** Reaction sequences obtained by lowering activation barriers (see Supplementary Material for tables and plots). The column “With Track 1: R2 + R6 + R8 + R10 + R11 + R3” indicates that a specific track is allowed to coexist with Track 1, such that both pathways have low barriers when running the simulation. The column “On its own” refers to a simulation in which only the track of interest is tested with low barriers; all other tracks have high barriers, including Track 1. The “✓” symbol indicates that the plot is the same as in Figure 7. The “~” symbol indicates that coupling occurs but at a much slower timescale. The “✗” indicates that coupling is lost.

Reaction Sequence		Pathway	With Track 1: R2 + R6 + R8 + R10 + R11+ R3	On Its Own
R2 R6 R8 R18 R20 R3	C + M → CM CM + A → AMC AMC + W → AMD + Z AMD + X → BMD + Y BMD → BM + D BM → B + M	Track 2 R2 + R6 + R8 + R18 + R20 + R3 C binds to M before A D is generated before B D is released before B	✓ Same overlap and timescale as Figure 7	✓ Same overlap and timescale as Figure 7
R1 R5 R7 R19 R17 R4	A + M → AM AM + C → AMC AMC + X → BMC + Y BMC + W → BMD + Z BMD → DM + B DM → D + M	Track 3 R1 + R5 + R7 + R19 + R17 + R4 A binds to M before C B is generated before D B is released before D	✗ Overlap loss Low D concentration	~ Much slower, 10^9^ s instead of 10^5^ s
R1 R5 R7 R19 R20 R3	A + M → AM AM + C → AMC AMC + X → BMC + Y BMC + W → BMD + Z BMD → BM + D BM → B + M	Track 4 R1 + R5 + R7 + R19 + R20 + R3 A binds to M before C B is generated before D D is released before B	✗ Overlap loss Low D concentration	~ Much slower, 10^9^ s instead of 10^5^ s
R1 R5 R7 R9 R12 R4	A + M → AM AM + C → AMC AMC + X → BMC + Y BMC → CM + B CM + W → DM + Z DM → D + M	Track 5 R1 + R5 + R7 + R9 + R12 + R4 A binds to M before C B is generated before D B is released before C is converted to D	✗ Overlap loss Low D concentration	~ Much slower, 10^10^ s instead of 10^5^ s
R1 R5 R8 R18 R17 R4	A + M → AM AM + C → AMC AMC + W → AMD + Z AMD + X → BMD + Y BMD → DM + B DM → D + M	Track 6 R1 + R5 + R8 + R18 + R17 + R4 A binds to M before C D is generated before B B is released before D	✗ Overlap loss Low D concentration	~ Much slower, 10^9^ s instead of 10^5^ s
R2 R6 R7 R19 R20 R3	C + M → CM CM + A → AMC AMC + X → BMC + Y BMC + W → BMD + Z BMD → BM + D BM → B + M	Track 7 R2 + R6 + R7 + R19 + R20 + R3 C binds to M before A B is generated before D D is released before B	~ Much slower, 10^7^ s instead of 10^5^ s	~ Much slower, 10^7^ s instead of 10^5^ s

Each track has an assigned color: Track 2 is purple, Track 3 is orange, Track 4 is pink, Track 5 is blue, Track 6 is light-blue, Track 7 is brown.

**Table 8:** List of all the reactions featured in Model-2, in which only Track 2 (highlighted in purple) has low activation barriers. Each reaction has a label in line with the diagram of Figure 6. All the reactions are listed in the direction of spontaneity from right to left (e.g., C + M ⇌ CM means conversion from C to CM is spontaneous, so its standard Gibbs free energy change, ΔG°, is negative). The forward and backward activation energy barriers are listed as E_a(forward)_ and E_a(backward)_. The forward and backward reaction rates (k, k_−1_) were calculated using Equation (3).

ID	Reaction	ΔG° (kcal mol^−1^)	E_a(forward)_ (kcal mol^−1^)	E_a(backward)_ (kcal mol^−1^)	k	k_−1_
R1	A + M ⇌ AM	−9	21	30	3.97 × 10^−16^	9.94 × 10^−23^
R2	C + M ⇌ CM	−1	0.1	1.1	8.45 × 10^−1^	1.56 × 10^−1^
R3	BM ⇌ B + M	−4	1	5	1.85 × 10^−1^	2.15 × 10^−4^
R4	DM ⇌ D + M	−5	25	30	4.62 × 10^−19^	9.94 × 10^−23^
R5	AMC ⇌ AM + C	−5	25	30	4.62 × 10^−19^	9.94 × 10^−23^
R6	CM + A ⇌ AMC	−3	1	4	1.85 × 10^−1^	1.17 × 10^−3^
R7	AMC + X ⇌ BMC + Y	−8	22	30	7.33 × 10^−17^	9.94 × 10^−23^
R8	AMC + W ⇌ AMD + Z	−2	0.1	2.1	8.45 × 10^−1^	2.88 × 10^−2^
R9	BMC ⇌ CM + B	−4	26	30	8.54 × 10^−20^	9.94 × 10^−23^
R10	AMD ⇌ AM + D	−1	29	30	5.38 × 10^−22^	9.94 × 10^−23^
R11	AM + X ⇌ BM + Y	−2	28	30	2.91 × 10^−21^	9.94 × 10^−23^
R12	DM + Z ⇌ CM + W	−8	22	30	7.33 × 10^−17^	9.94 × 10^−23^
R13	BM + C ⇌ CM + B	−5	25	30	4.62 × 10^−19^	9.94 × 10^−23^
R14	DM + A ⇌ AM + D	−14	16	30	1.84 × 10^−12^	9.94 × 10^−23^
R15	BM + C ⇌ BMC	−1	29	30	5.38 × 10^−22^	9.94 × 10^−23^
R16	DM + A ⇌ AMD	−13	17	30	3.40 × 10^−13^	9.94 × 10^−23^
R17	DM + B ⇌ BMD	0	30	30	9.94 × 10^−23^	9.94 × 10^−23^
R18	AMD + X ⇌ BMD + Y	−2	0.2	2.2	7.13 × 10^−1^	2.43 × 10^−2^
R19	BMD + Z ⇌ BMC + W	−4	26	30	8.54 × 10^−20^	9.94 × 10^−23^
R20	BMD ⇌ BM + D	−1	0.1	1.1	8.45 × 10^−1^	1.56 × 10^−1^

**Table 9:** Assigned standard Gibbs free energies of the species featured in Model-3. These values were arbitrarily chosen under the condition that ΔG°_A-to-B_ = −19 kcal mol^−1^ and the ΔG°_C-to-D_ = +2 kcal mol^−1^, regardless of which intermediates were used to achieve A-to-B and C-to-D conversions.

Species	G° (kcal mol^−1^)
A	20
B	1
C	5
D	7
M	0
X	0
Y	0
W	0
Z	0
AM	11
BM	9
CM	4
DM	8
AMC	14
BMC	13
AMD	19
BMD	10

**Table 10:** List of all the reactions featured in Model-3, with the reaction steps of Track 3 highlighted in orange. Each reaction has a label in line with the diagram of Figure 13. All the reactions are listed in the direction of spontaneity from right to left (e.g., A + M ⇌ AM means conversion from A to AM is spontaneous, so its standard Gibbs free energy change, ΔG°, is negative). The forward and backward activation energy barriers are listed as E_a(forward)_ and E_a(backward)_. The forward and backward reaction rates (k, k_−1_) are calculated using Equation (3).

ID	Reaction	ΔG° (kcal mol^−1^)	E_a(forward)_ (kcal mol^−1^)	E_a(backward)_ (kcal mol^−1^)	k	k_−1_
R1	A + M ⇌ AM	−9	0.1	9.1	8.45 × 10^−1^	2.12 × 10^−7^
R2	C + M ⇌ CM	−1	29	30	5.38 × 10^−22^	9.94 × 10^−23^
R3	BM ⇌ B + M	−8	22	30	7.33 × 10^−17^	9.94 × 10^−23^
R4	DM ⇌ D + M	−1	0.1	1.1	8.45 × 10^−1^	1.56 × 10^−1^
R5	AM + C ⇌ AMC	−2	0.1	2.1	8.45 × 10^−1^	2.88 × 10^−2^
R6	CM + A ⇌ AMC	−10	20	30	2.15 × 10^−15^	9.94 × 10^−23^
R7	AMC + X ⇌ BMC + Y	−1	0.1	1.1	8.45 × 10^−1^	1.56 × 10^−1^
R8	AMD + Z ⇌ AMC + W	−5	25	30	4.62 × 10^−19^	9.94 × 10^−23^
R9	BMC ⇌ CM + B	−8	22	30	7.33 × 10^−17^	9.94 × 10^−23^
R10	AMD ⇌ AM + D	−1	29	30	5.38 × 10^−22^	9.94 × 10^−23^
R11	AM + X ⇌ BM + Y	−2	28	30	2.91 × 10^−21^	9.94 × 10^−23^
R12	DM + Z ⇌ CM + W	−4	26	30	8.54 × 10^−20^	9.94 × 10^−23^
R13	BM + C ⇌ CM + B	−9	21	30	3.97 × 10^−16^	9.94 × 10^−23^
R14	DM + A ⇌ AM + D	−10	20	30	2.15 × 10^−15^	9.94 × 10^−23^
R15	BM + C ⇌ BMC	−1	29	30	5.38 × 10^−22^	9.94 × 10^−23^
R16	DM + A ⇌ AMD	−9	21	30	3.97 × 10^−16^	9.94 × 10^−23^
R17	BMD ⇌ DM + B	−1	0.1	1.1	8.45 × 10^−1^	1.56 × 10^−1^
R18	AMD + X ⇌ BMD + Y	−9	21	30	3.97 × 10^−16^	9.94 × 10^−23^
R19	BMC + W ⇌ BMD + Z	−3	1.1	4.1	1.56 × 10^−1^	9.84 × 10^−4^
R20	BM + D ⇌ BMD	−6	24	30	2.50 × 10^−18^	9.94 × 10^−23^

## Data Availability

Data are contained within the article and Supplementary Materials.

## Supplementary Materials

The following supporting information can be downloaded at: https://www.mdpi.com/article/10.3390/molecules30040911/s1, Figures S1–S16: Figure S1: Data, network and plot of Model-0. (a) G° values for the network used to study Model-0. (b) The network of Model-0. (c) Table of values for the reactions featured in the network. (d) The resulting plot. Figure S2: Data, network and plot of Model-0-B. (a) G° values for the network used to study Model-0-B. (b) The network of Model-0-B. (c) Table of values for the reactions featured in the network. (d) The resulting plot. Figure S3: Data, network and plot of Track-1 (R2 + R6 + R8 + R10 + R11+ R3). (a) G° values for the network used to study Track-1. (b) The network, with Track-1 highlighted in green. (c) Table of values for the reactions featured in the network, with the reaction steps of Track-1 highlighted in green. (d) The resulting plot, which is called Model-1. Figure S4: Data, network and plot of Track-1 (R2 + R6 + R8 + R10 + R11+ R3) and Track-2 (R2 + R6 + R8 + R18 + R20 + R3). (a) G° values for the network used to study Track-1 and Track-2. (b) The network, with Track-1 highlighted in green and Track-2 highlighted in purple. (c) Table of values for the reactions featured in the network, with the reaction steps of Track-1 highlighted in green and the ones of Track-2 highlighted in purple (reaction steps shared by Track-1 and Track-2 are highlighted in purple). (d) The resulting plot. Figure S5: Data, network and plot of Track-2 (R2 + R6 + R8 + R18 + R20 + R3). (a) G° values for the network used to study Track-2. (b) The network, with Track-2 highlighted in purple. (c) Table of values for the reactions featured in the network, with the reaction steps of Track-2 highlighted in purple. (d) The resulting plot, which is called Model-2. Figure S6: Data, network and plot of Track-1 (R2 + R6 + R8 + R10 + R11+ R3) and Track-3 (R1 + R5 + R7 + R19 + R17 + R4). (a) G° values for the network used to study Track-1 and Track-3. (b) The network, with Track-1 highlighted in green and Track-3 highlighted in orange. (c) Table of values for the reactions featured in the network, with the reaction steps of Track-1 highlighted in green and the ones of Track-3 highlighted in orange. (d) The resulting plot. Figure S7: Data, network and plot of Track-3 (R1 + R5 + R7 + R19 + R17 + R4). (a) G° values for the network used to study Track-3. (b) The network, with Track-3 highlighted in orange. (c) Table of values for the reactions featured in the network, with the reaction steps of Track-3 highlighted in orange. (d) The resulting plot. Figure S8: Data, network and plot of Track-1 (R2 + R6 + R8 + R10 + R11+ R3) and Track-4 (R1 + R5 + R7 + R19 + R20 + R3). (a) G° values for the network used to study Track-1 and Track-4. (b) The network, with Track-1 highlighted in green and Track-4 highlighted in pink. (c) Table of values for the reactions featured in the network, with the reaction steps of Track-1 highlighted in green and the ones of Track-4 highlighted in pink (R3 is shared by Track-1 and Track-4 and is highlighted in pink). (d) The resulting plot. Figure S9: Data, network and plot of Track-4. (a) G° values for the network used to study Track-4 (R1 + R5 + R7 + R19 + R20 + R3). (b) The network, with Track-4 highlighted in pink. (c) Table of values for the reactions featured in the network, with the reaction steps of Track-4 highlighted in pink. (d) The resulting plot. Figure S10: Data, network and plot of Track-1 (R2 + R6 + R8 + R10 + R11+ R3) and Track-5 (R1 + R5 + R7 + R9 + R12 + R4). (a) G° values for the network used to study Track-1 and Track-5. (b) The network, with Track-1 highlighted in green and Track-5 highlighted in blue. (c) Table of values for the reactions featured in the network, with the reaction steps of Track-1 highlighted in green and the ones of Track-5 highlighted in blue. (d) The resulting plot. Figure S11: Data, network and plot of Track-5 (R1 + R5 + R7 + R9 + R12 + R4). (a) G° values for the network used to study Track-5. (b) The network, with Track-5 highlighted in blue. (c) Table of values for the reactions featured in the network, with the reaction steps of Track-5 highlighted in blue. (d) The resulting plot. Figure S12: Data, network and plot of Track-1 (R2 + R6 + R8 + R10 + R11+ R3) and Track-6 (R1 + R5 + R8 + R18 + R17 + R4). (a) G° values for the network used to study Track-1 and Track-6. (b) The network, with Track-1 highlighted in green and Track-6 highlighted in light-blue. (c) Table of values for the reactions featured in the network, with the reaction steps of Track-1 highlighted in green and the ones of Track-6 highlighted in light-blue (R8 is shared by Track-1 and Track-6 and is highlighted in light-blue). (d) The resulting plot. Figure S13: Data, network and plot of Track-6 (R1 + R5 + R8 + R18 + R17 + R4). (a) G° values for the network used to study Track-6. (b) The network, with Track-6 highlighted in light-blue. (c) Table of values for the reactions featured in the network, with the reaction steps of Track-6 highlighted in light-blue. (d) The resulting plot. Figure S14: Data, network and plot of Track-1 (R2 + R6 + R8 + R10 + R11+ R3) and Track-7 (R2 + R6 + R7 + R19 + R20 + R3). (a) G° values for the network used to study Track-1 and Track-7. (b) The network, with Track-1 highlighted in green and Track-7 highlighted in brown. (c) Table of values for the reactions featured in the network, with the reaction steps of Track-1 highlighted in green and the ones of Track-7 highlighted in brown (reaction steps shared by Track-1 and Track-7 are highlighted in brown). (d) The resulting plot. Figure S15: Data, network and plot of Track-7 (R2 + R6 + R7 + R19 + R20 + R3). (a) G° values for the network used to study Track-7. (b) The network, with Track-7 highlighted in brown. (c) Table of values for the reactions featured in the network, with the reaction steps of Track-7 highlighted in brown. (d) The resulting plot. Figure S16: Data, network and plot of Model-3. This model relies on the reaction steps featured in Track-3, but the reaction steps in Model-3 have different directionalities compared to the ones seen in Figures S6 and S7, because Model-3 has different G° values assigned to it. (a) G° values for the network used to study Model-3. (b) The network, with Model-3 highlighted in orange. (c) Table of values for the reactions featured in the network, with the reaction steps of Model-3 highlighted in orange. (d) The resulting plot.

## References

[1] Deffner S., Bonança M.V.S. (2020). Thermodynamic control—An old paradigm with new applications. EPL.

[2] Marsden S.R., Mestrom L., McMillan D.G.G., Hanefeld U. (2020). Thermodynamically and Kinetically Controlled Reactions in Biocatalysis—From Concepts to Perspectives. ChemCatChem.

[3] Astumian R.D. (2019). Kinetic asymmetry allows macromolecular catalysts to drive an information ratchet. Nat. Commun..

[4] Borsley S., Gallagher J.M., Leigh D.A., Roberts B.M.W. (2023). Ratcheting synthesis. Nat. Rev. Chem..

[5] Sangchai T., Al Shehimy S., Penocchio E., Ragazzon G. (2023). Artificial Molecular Ratchets: Tools Enabling Endergonic Processes. Angew. Chem. Int. Ed..

[6] Aprahamian I., Goldup S.M. (2023). Non-equilibrium Steady States in Catalysis, Molecular Motors, and Supramolecular Materials: Why Networks and Language Matter. J. Am. Chem. Soc..

[7] Olivieri E., Gallagher J.M., Betts A., Mrad T.W., Leigh D.A. (2024). Endergonic synthesis driven by chemical fuelling. Nat. Synth..

[8] Branscomb E., Russell M.J. (2013). Turnstiles and bifurcators: The disequilibrium converting engines that put metabolism on the road. Biochim. Biophys. Acta (BBA)-Bioenerg..

[9] Astumian R.D., Mukherjee S., Warshel A. (2016). The Physics and Physical Chemistry of Molecular Machines. ChemPhysChem.

[10] Shore D., Albert B. (2022). Ribosome biogenesis and the cellular energy economy. Curr. Biol..

[11] Borsley S., Leigh D.A., Roberts B.M.W. (2024). Molecular Ratchets and Kinetic Asymmetry: Giving Chemistry Direction. Angew. Chem. Int. Ed..

[12] Ragazzon G., Prins L.J. (2018). Energy consumption in chemical fuel-driven self-assembly. Nat. Nanotechbol..

[13] Amano S., Borsley S., Leigh D.A., Sun Z. (2021). Chemical engines: Driving systems away from equilibrium through catalyst reaction cycles. Nat. Nanotechnol..

[14] Astumian R.D., Pezzato C., Feng Y., Qiu Y., McGonigal P.R., Cheng C., Stoddart J.F. (2020). Non-equilibrium kinetics and trajectory thermodynamics of synthetic molecular pumps. Mater. Chem. Front..

[15] Biagini C., Di Stefano S. (2020). Abiotic Chemical Fuels for the Operation of Molecular Machines. Angew. Chem. Int. Ed..

[16] Brown A.I., Sivak D.A. (2020). Theory of Nonequilibrium Free Energy Transduction by Molecular Machines. Chem. Rev..

[17] Binks L., Borsley S., Gingrich T.R., Leigh D.A., Penocchio E., Roberts B.M.W. (2023). The role of kinetic asymmetry and power strokes in an information ratchet. Chem.

[18] Amano S., Esposito M., Kreidt E., Leigh D.A., Penocchio E., Roberts B.M.W. (2022). Using Catalysis to Drive Chemistry Away from Equilibrium: Relating Kinetic Asymmetry, Power Strokes, and the Curtin–Hammett Principle in Brownian Ratchets. J. Am. Chem. Soc..

[19] Branscomb E., Biancalani T., Goldenfeld N., Russell M. (2017). Escapement mechanisms and the conversion of disequilibria; the engines of creation. Phys. Rep..

[20] Borsley S., Leigh D.A., Roberts B.M.W. (2021). A Doubly Kinetically-Gated Information Ratchet Autonomously Driven by Carbodiimide Hydration. J. Am. Chem. Soc..

[21] Borsley S., Leigh D.A., Roberts B.M.W., Vitorica-Yrezabal I.J. (2022). Tuning the Force, Speed, and Efficiency of an Autonomous Chemically Fueled Information Ratchet. J. Am. Chem. Soc..

[22] Tkačik G., Bialek W. (2016). Information Processing in Living Systems. Annu. Rev. Condens. Matter Phys..

[23] Mizraji E. (2021). The biological Maxwell’s demons: Exploring ideas about the information processing in biological systems. Theory Biosci..

[24] Efremov A., Wang Z. (2011). Universal optimal working cycles of molecular motors. Phys. Chem. Chem. Phys..

[25] COPASI. https://copasi.org/.

