# Supplementary material for: Computational Analysis of the Kinetic Requirements for Coupled Reaction Systems
Source: Molecules. 2025 Feb 15;30(4):911. doi: 10.3390/molecules30040911 (PMC11858731; doi:10.3390/molecules30040911)
Supplement: Supplementary file 1 [file molecules-30-00911-s001.zip › molecules-3396470-supplementary.pdf]

SUPPLEMENTARY MATERIALS

Computational Analysis of the Kinetic Requirements for Coupled Reaction Systems

Sara Incarbone * and Luca De Gioia *

Department of Biotechnology and Biosciences, University of Milan-Bicocca, Piazza della Scienza 2, 20126 Milan, Italy
* Correspondence: s.incarbone@campus.unimib.it (S.I.); luca.degioia@unimib.it (L.D.G.)

(a)

Species	G° (kcal mol⁻¹)
A	10
B	5
C	5
D	7
M	0
X	0
Y	0
W	0
Z	0
AM	8
BM	9
CM	13
DM	12
AMC	15
BMC	13
AMD	18
BMD	17
**Overall Reaction**	**ΔG° (kcal mol⁻¹)**
A → B	−5
C → B	+2

(b)

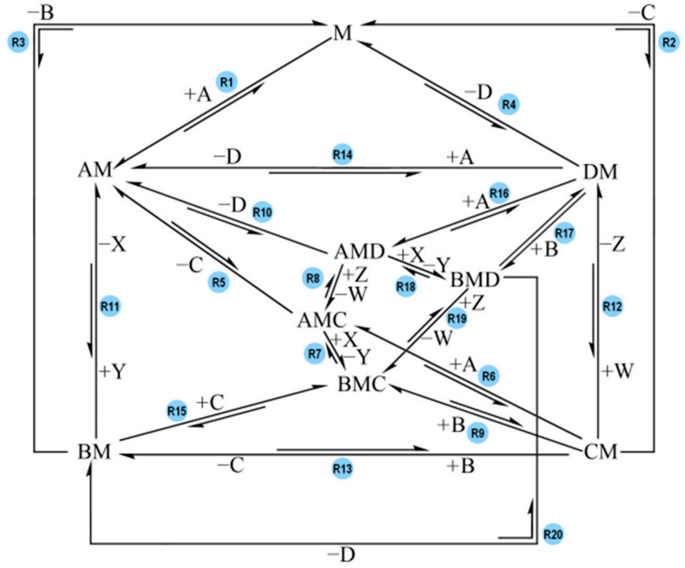

(c)

ID	Reaction	ΔG° (kcal mol⁻¹)	$E_{a(forward)}$ (kcal mol⁻¹)	$E_{a(backward)}$ (kcal mol⁻¹)	k	k_{-1}
R1	A + M ⇌ AM	−2	4	6	1.17×10^{-3}	3.98×10^{-5}
R2	CM ⇌ C + M	−8	1	9	1.85×10^{-1}	2.51×10^{-7}
R3	BM ⇌ B + M	−4	1	5	1.85×10^{-1}	2.15×10^{-4}
R4	DM ⇌ D + M	−5	2	7	3.41×10^{-2}	7.35×10^{-6}
R5	AMC ⇌ AM + C	−2	2	4	3.41×10^{-2}	1.17×10^{-3}
R6	CM + A ⇌ AMC	−8	2	10	3.41×10^{-2}	4.63×10^{-8}
R7	AMC + X ⇌ BMC + Y	−2	0.1	2.1	8.45×10^{-1}	2.88×10^{-2}
R8	AMD + Z ⇌ AMC + W	−3	0.1	3.1	8.45×10^{-1}	5.33×10^{-3}
R9	CM + B ⇌ BMC	−5	10	15	4.63×10^{-8}	9.97×10^{-12}
R10	AMD ⇌ AM + D	−3	12	15	1.58×10^{-9}	9.97×10^{-12}
R11	BM + Y ⇌ AM + X	−1	14	15	5.40×10^{-11}	9.97×10^{-12}
R12	CM + W ⇌ DM + Z	−1	14	15	5.40×10^{-11}	9.97×10^{-12}
R13	CM + B ⇌ BM + C	−4	11	15	8.56×10^{-9}	9.97×10^{-12}
R14	DM + A ⇌ AM + D	−7	8	15	1.36×10^{-6}	9.97×10^{-12}
R15	BM + C ⇌ BMC	−1	14	15	5.40×10^{-11}	9.97×10^{-12}
R16	DM + A ⇌ AMD	−4	11	15	8.56×10^{-9}	9.97×10^{-12}
R17	DM + B ⇌ BMD	0	3	3	6.31×10^{-3}	6.31×10^{-3}
R18	AMD + X ⇌ BMD + Y	−1	0.1	1.1	8.45×10^{-1}	1.56×10^{-1}
R19	BMD + Z ⇌ BMC + W	−4	0.1	4.1	8.45×10^{-1}	9.84×10^{-4}
R20	BMD ⇌ BM + D	−1	0.1	1.1	8.45×10^{-1}	1.56×10^{-1}

(d)

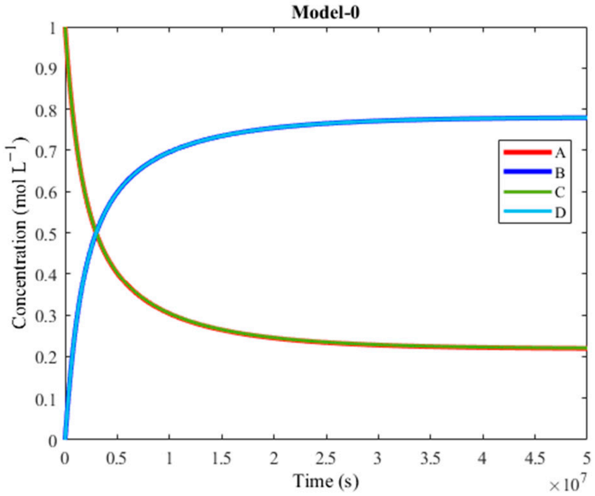

Figure S1. Data, network and plot of Model-0. (**a**) G° values for the network used to study Model-0. (**b)** The network of Model-0. (**c**) Table of values for the reactions featured in the network. (**d**) The resulting plot.

(a)

Species	G° (kcal mol⁻¹)
A	10
B	5
C	5
D	7
M	0
X	0
Y	0
W	0
Z	0
AM	8
BM	9
CM	13
DM	12
AMC	15
BMC	13
AMD	18
BMD	17
**Overall Reaction**	**ΔG° (kcal mol⁻¹)**
A → B	−5
C → B	+2

(b)

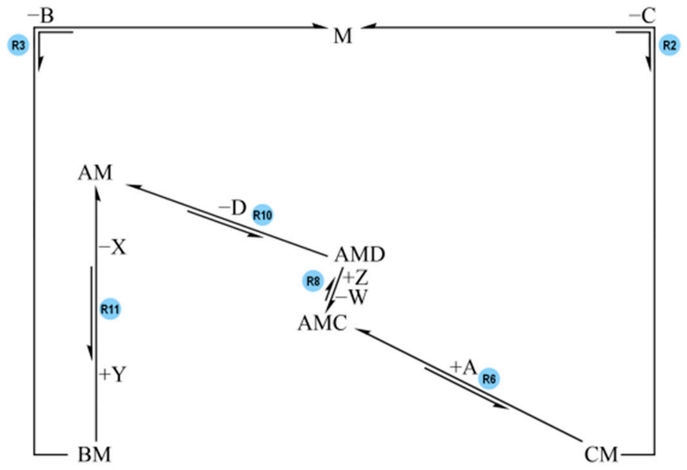

(c)

ID	Reaction	ΔG° (kcal mol⁻¹)	$E_{a(forward)}$ (kcal mol⁻¹)	$E_{a(backward)}$ (kcal mol⁻¹)	k	k_{-1}
R2	CM ⇌ C + M	−8	1	9	1.85×10^{-1}	2.51×10^{-7}
R3	BM ⇌ B + M	−4	1	5	1.85×10^{-1}	2.15×10^{-4}
R6	CM + A ⇌ AMC	−8	2	10	3.41×10^{-2}	4.63×10^{-8}
R8	AMD + Z ⇌ AMC + W	−3	0.1	3.1	8.45×10^{-1}	5.33×10^{-3}
R10	AMD ⇌ AM + D	−3	4	7	1.17×10^{-3}	7.35×10^{-6}
R11	BM + Y ⇌ AM + X	−1	1	2	1.85×10^{-1}	3.41×10^{-2}

(d)

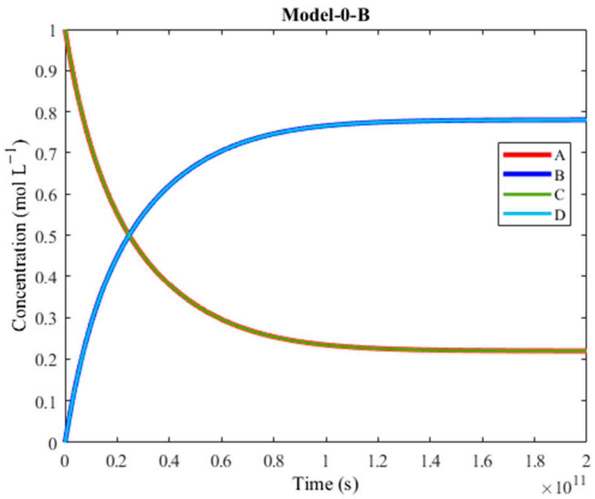

Figure S2. Data, network and plot of Model-0-B. (**a**) G° values for the network used to study Model-0-B. (**b**) The network of Model-0-B. (**c**) Table of values for the reactions featured in the network. (**d**) The resulting plot.

(a)

Species	G° (kcal mol⁻¹)
A	20
B	5
C	5
D	7
M	0
X	0
Y	0
W	0
Z	0
AM	11
BM	9
CM	4
DM	12
AMC	21
BMC	13
AMD	19
BMD	17
**Overall Reaction**	**ΔG° (kcal mol⁻¹)**
A → B	−15
C → B	+2

(b)

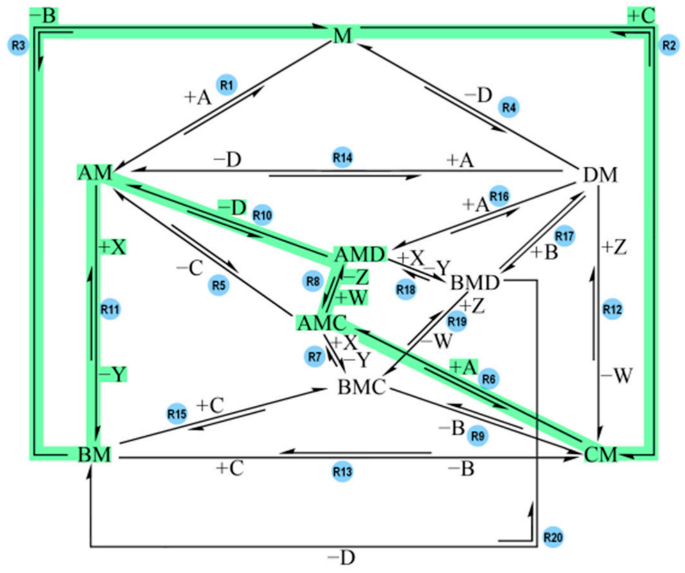

(c)

ID	Reaction	ΔG° (kcal mol⁻¹)	$E_{a(forward)}$ (kcal mol⁻¹)	$E_{a(backward)}$ (kcal mol⁻¹)	k	k_{-1}
R1	A + M ⇌ AM	−9	21	30	3.97×10^{-16}	9.94×10^{-23}
R2	C + M ⇌ CM	−1	0.1	1.1	8.45×10^{-1}	1.56×10^{-1}
R3	BM ⇌ B + M	−4	1	5	1.85×10^{-1}	2.15×10^{-4}
R4	DM ⇌ D + M	−5	25	30	4.62×10^{-19}	9.94×10^{-23}
R5	AMC ⇌ AM + C	−5	25	30	4.62×10^{-19}	9.94×10^{-23}
R6	CM + A ⇌ AMC	−3	1	4	1.85×10^{-1}	1.17×10^{-3}
R7	AMC + X ⇌ BMC + Y	−8	22	30	7.33×10^{-17}	9.94×10^{-23}
R8	AMC + W ⇌ AMD + Z	−2	0.1	2.1	8.45×10^{-1}	2.88×10^{-2}
R9	BMC ⇌ CM + B	−4	26	30	8.54×10^{-20}	9.94×10^{-23}
R10	AMD ⇌ AM + D	−1	1	2	1.85×10^{-1}	3.41×10^{-2}
R11	AM + X ⇌ BM + Y	−2	0.1	2.1	8.45×10^{-1}	2.88×10^{-2}
R12	DM + Z ⇌ CM + W	−8	22	30	7.33×10^{-17}	9.94×10^{-23}
R13	BM + C ⇌ CM + B	−5	25	30	4.62×10^{-19}	9.94×10^{-23}
R14	DM + A ⇌ AM + D	−14	16	30	1.84×10^{-12}	9.94×10^{-23}
R15	BM + C ⇌ BMC	−1	29	30	5.38×10^{-22}	9.94×10^{-23}
R16	DM + A ⇌ AMD	−13	17	30	3.40×10^{-13}	9.94×10^{-23}
R17	DM + B ⇌ BMD	0	30	30	9.94×10^{-23}	9.94×10^{-23}
R18	AMD + X ⇌ BMD + Y	−2	28	30	2.91×10^{-21}	9.94×10^{-23}
R19	BMD + Z ⇌ BMC + W	−4	26	30	8.54×10^{-20}	9.94×10^{-23}
R20	BMD ⇌ BM + D	−1	29	30	5.38×10^{-22}	9.94×10^{-23}

(d)

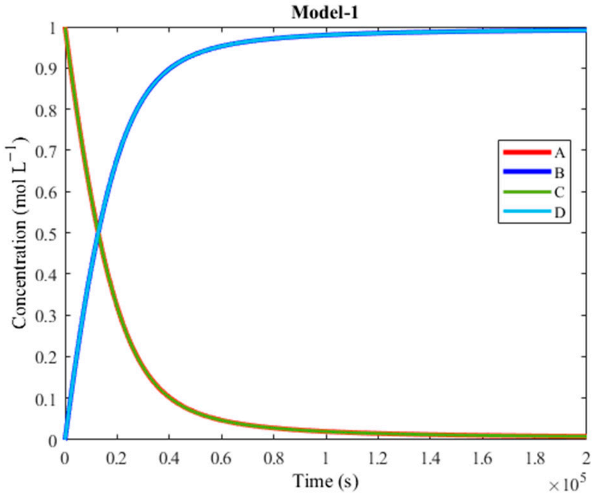

Figure S3. Data, network and plot of Track-1 (R2 + R6 + R8 + R10 + R11+ R3). (**a**) G° values for the network used to study Track-1. (**b)** The network, with Track-1 highlighted in green. (**c**) Table of values for the reactions featured in the network, with the reaction steps of Track-1 highlighted in green. (**d**) The resulting plot, which is called Model-1.

(a)

Species	G° (kcal mol⁻¹)
A	20
B	5
C	5
D	7
M	0
X	0
Y	0
W	0
Z	0
AM	11
BM	9
CM	4
DM	12
AMC	21
BMC	13
AMD	19
BMD	17
**Overall Reaction**	**ΔG° (kcal mol⁻¹)**
A → B	−15
C → B	+2

(b)

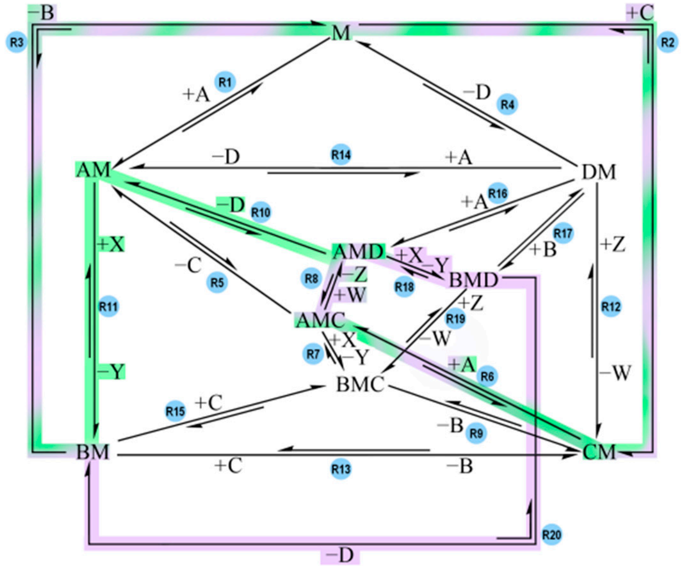

(c)

ID	Reaction	ΔG° (kcal mol⁻¹)	$E_{a(forward)}$ (kcal mol⁻¹)	$E_{a(backward)}$ (kcal mol⁻¹)	k	k_{-1}
R1	A + M ⇌ AM	−9	21	30	3.97×10^{-16}	9.94×10^{-23}
R2	C + M ⇌ CM	−1	0.1	1.1	8.45×10^{-1}	1.56×10^{-1}
R3	BM ⇌ B + M	−4	1	5	1.85×10^{-1}	2.15×10^{-4}
R4	DM ⇌ D + M	−5	25	30	4.62×10^{-19}	9.94×10^{-23}
R5	AMC ⇌ AM + C	−5	25	30	4.62×10^{-19}	9.94×10^{-23}
R6	CM + A ⇌ AMC	−3	1	4	1.85×10^{-1}	1.17×10^{-3}
R7	AMC + X ⇌ BMC + Y	−8	22	30	7.33×10^{-17}	9.94×10^{-23}
R8	AMC + W ⇌ AMD + Z	−2	0.1	2.1	8.45×10^{-1}	2.88×10^{-2}
R9	BMC ⇌ CM + B	−4	26	30	8.54×10^{-20}	9.94×10^{-23}
R10	AMD ⇌ AM + D	−1	1	2	1.85×10^{-1}	3.41×10^{-2}
R11	AM + X ⇌ BM + Y	−2	0.1	2.1	8.45×10^{-1}	2.88×10^{-2}
R12	DM + Z ⇌ CM + W	−8	22	30	7.33×10^{-17}	9.94×10^{-23}
R13	BM + C ⇌ CM + B	−5	25	30	4.62×10^{-19}	9.94×10^{-23}
R14	DM + A ⇌ AM + D	−14	16	30	1.84×10^{-12}	9.94×10^{-23}
R15	BM + C ⇌ BMC	−1	29	30	5.38×10^{-22}	9.94×10^{-23}
R16	DM + A ⇌ AMD	−13	17	30	3.40×10^{-13}	9.94×10^{-23}
R17	DM + B ⇌ BMD	0	30	30	9.94×10^{-23}	9.94×10^{-23}
R18	AMD + X ⇌ BMD + Y	−2	0.2	2.2	7.13×10^{-1}	2.43×10^{-2}
R19	BMD + Z ⇌ BMC + W	−4	26	30	8.54×10^{-20}	9.94×10^{-23}
R20	BMD ⇌ BM + D	−1	0.1	1.1	8.45×10^{-1}	1.56×10^{-1}

(d)

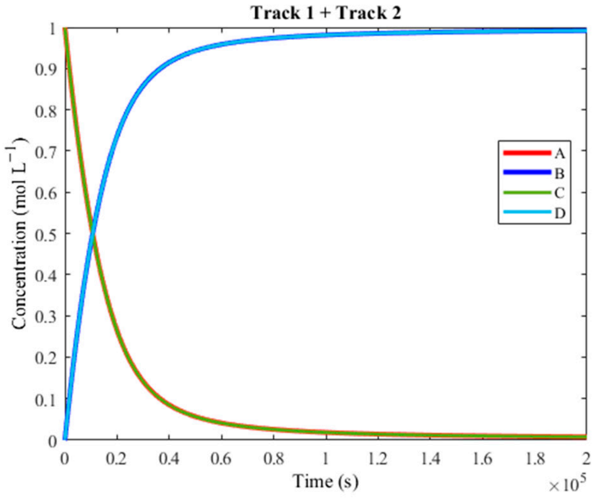

Figure S4. Data, network and plot of Track-1 (R2 + R6 + R8 + R10 + R11+ R3) and Track-2 (R2 + R6 + R8 + R18 + R20 + R3). (**a**) G° values for the network used to study Track-1 and Track-2. (**b**) The network, with Track-1 highlighted in green and Track-2 highlighted in purple. (**c**) Table of values for the reactions featured in the network, with the reaction steps of Track-1 highlighted in green and the ones of Track-2 highlighted in purple (reaction steps shared by Track-1 and Track-2 are highlighted in purple). (**d**) The resulting plot.

(a)

Species	G° (kcal mol⁻¹)
A	20
B	5
C	5
D	7
M	0
X	0
Y	0
W	0
Z	0
AM	11
BM	9
CM	4
DM	12
AMC	21
BMC	13
AMD	19
BMD	17
**Overall Reaction**	**ΔG° (kcal mol⁻¹)**
A → B	–15
C → B	+2

(b)

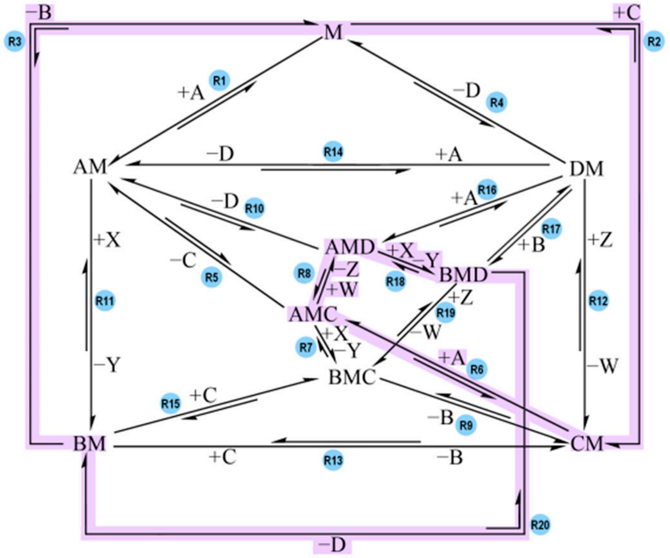

(c)

ID	Reaction	ΔG° (kcal mol⁻¹)	$E_{a(forward)}$ (kcal mol⁻¹)	$E_{a(backward)}$ (kcal mol⁻¹)	k	k_{-1}
R1	A + M ⇌ AM	–9	21	30	3.97×10^{-16}	9.94×10^{-23}
R2	C + M ⇌ CM	–1	0.1	1.1	8.45×10^{-1}	1.56×10^{-1}
R3	BM ⇌ B + M	–4	1	5	1.85×10^{-1}	2.15×10^{-4}
R4	DM ⇌ D + M	–5	25	30	4.62×10^{-19}	9.94×10^{-23}
R5	AMC ⇌ AM + C	–5	25	30	4.62×10^{-19}	9.94×10^{-23}
R6	CM + A ⇌ AMC	–3	1	4	1.85×10^{-1}	1.17×10^{-3}
R7	AMC + X ⇌ BMC + Y	–8	22	30	7.33×10^{-17}	9.94×10^{-23}
R8	AMC + W ⇌ AMD + Z	–2	0.1	2.1	8.45×10^{-1}	2.88×10^{-2}
R9	BMC ⇌ CM + B	–4	26	30	8.54×10^{-20}	9.94×10^{-23}
R10	AMD ⇌ AM + D	–1	29	30	5.38×10^{-22}	9.94×10^{-23}
R11	AM + X ⇌ BM + Y	–2	28	30	2.91×10^{-21}	9.94×10^{-23}
R12	DM + Z ⇌ CM + W	–8	22	30	7.33×10^{-17}	9.94×10^{-23}
R13	BM + C ⇌ CM + B	–5	25	30	4.62×10^{-19}	9.94×10^{-23}
R14	DM + A ⇌ AM + D	–14	16	30	1.84×10^{-12}	9.94×10^{-23}
R15	BM + C ⇌ BMC	–1	29	30	5.38×10^{-22}	9.94×10^{-23}
R16	DM + A ⇌ AMD	–13	17	30	3.40×10^{-13}	9.94×10^{-23}
R17	DM + B ⇌ BMD	0	30	30	9.94×10^{-23}	9.94×10^{-23}
R18	AMD + X ⇌ BMD + Y	–2	0.2	2.2	7.13×10^{-1}	2.43×10^{-2}
R19	BMD + Z ⇌ BMC + W	–4	26	30	8.54×10^{-20}	9.94×10^{-23}
R20	BMD ⇌ BM + D	–1	0.1	1.1	8.45×10^{-1}	1.56×10^{-1}

(d)

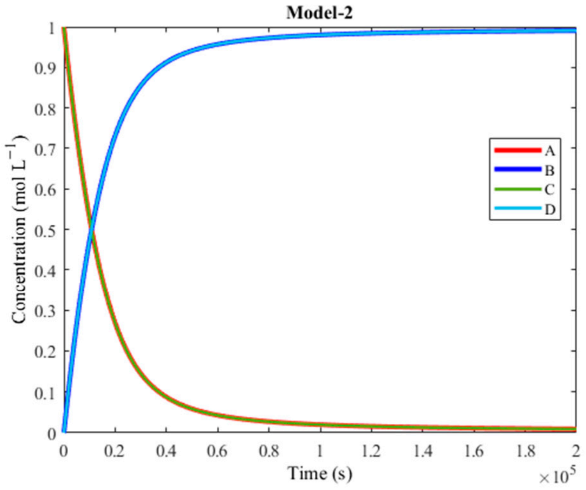

Figure S5. Data, network and plot of Track-2 (R2 + R6 + R8 + R18 + R20 + R3). (**a**) G° values for the network used to study Track-2. (**b**) The network, with Track-2 highlighted in purple. (**c**) Table of values for the reactions featured in the network, with the reaction steps of Track-2 highlighted in purple. (**d**) The resulting plot, which is called Model-2.

(a)

Species	G° (kcal mol^{-1})
A	20
B	5
C	5
D	7
M	0
X	0
Y	0
W	0
Z	0
AM	11
BM	9
CM	4
DM	12
AMC	21
BMC	13
AMD	19
BMD	17
**Overall Reaction**	**ΔG° (kcal mol^{-1})**
A → B	–15
C → B	+2

(b)

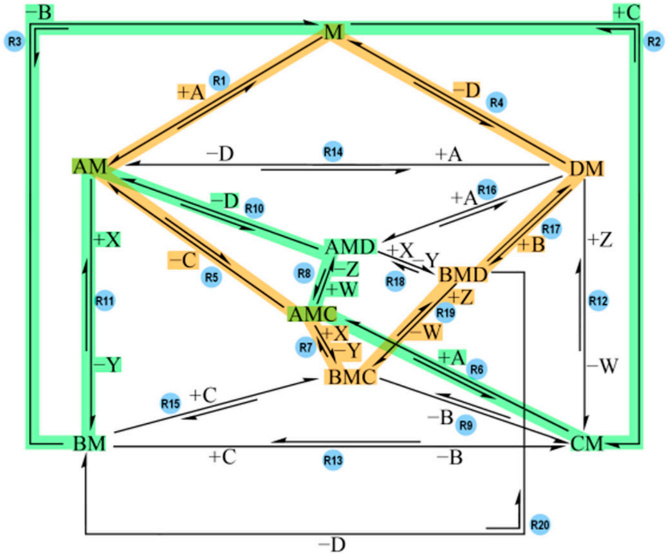

(c)

ID	Reaction	ΔG° (kcal mol^{-1})	$E_{a(forward)}$ (kcal mol^{-1})	$E_{a(backward)}$ (kcal mol^{-1})	k	k_{-1}
R1	A + M ⇌ AM	−9	0.1	9.1	8.45×10^{-1}	2.12×10^{-7}
R2	C + M ⇌ CM	−1	0.1	1.1	8.45×10^{-1}	1.56×10^{-1}
R3	BM ⇌ B + M	−4	1	5	1.85×10^{-1}	2.15×10^{-4}
R4	DM ⇌ D + M	−5	0.1	5.1	8.45×10^{-1}	1.82×10^{-4}
R5	AMC ⇌ AM + C	−5	0.1	5.1	8.45×10^{-1}	1.82×10^{-4}
R6	CM + A ⇌ AMC	−3	1	4	1.85×10^{-1}	1.17×10^{-3}
R7	AMC + X ⇌ BMC + Y	−8	0.1	8.1	8.45×10^{-1}	1.15×10^{-6}
R8	AMC + W ⇌ AMD + Z	−2	0.1	2.1	8.45×10^{-1}	2.88×10^{-2}
R9	BMC ⇌ CM + B	−4	26	30	8.54×10^{-20}	9.94×10^{-23}
R10	AMD ⇌ AM + D	−1	1	2	1.85×10^{-1}	3.41×10^{-2}
R11	AM + X ⇌ BM + Y	−2	0.1	2.1	8.45×10^{-1}	2.88×10^{-2}
R12	DM + Z ⇌ CM + W	−8	22	30	7.33×10^{-17}	9.94×10^{-23}
R13	BM + C ⇌ CM + B	−5	25	30	4.62×10^{-19}	9.94×10^{-23}
R14	DM + A ⇌ AM + D	−14	16	30	1.84×10^{-12}	9.94×10^{-23}
R15	BM + C ⇌ BMC	−1	29	30	5.38×10^{-22}	9.94×10^{-23}
R16	DM + A ⇌ AMD	−13	17	30	3.40×10^{-13}	9.94×10^{-23}
R17	DM + B ⇌ BMD	0	1	1	1.85×10^{-1}	1.85×10^{-1}
R18	AMD + X ⇌ BMD + Y	−2	28	30	2.91×10^{-21}	9.94×10^{-23}
R19	BMD + Z ⇌ BMC + W	−4	0.1	4.1	8.45×10^{-1}	9.84×10^{-4}
R20	BMD ⇌ BM + D	−1	29	30	5.38×10^{-22}	9.94×10^{-23}

(d)

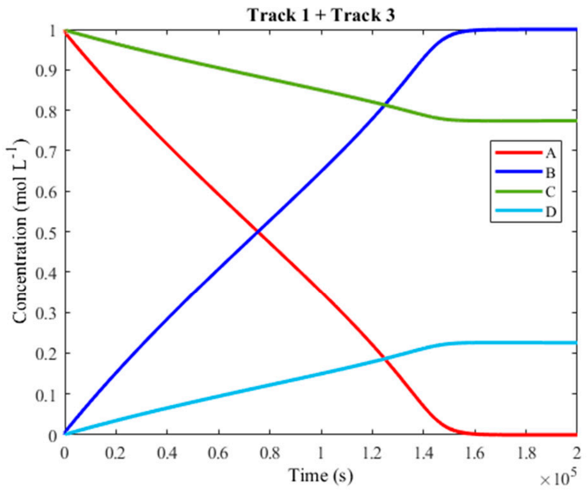

Figure S6. Data, network and plot of Track-1 (R2 + R6 + R8 + R10 + R11+ R3) and Track-3 (R1 + R5 + R7 + R19 + R17 + R4). (**a**) G° values for the network used to study Track-1 and Track-3. (**b**) The network, with Track-1 highlighted in green and Track-3 highlighted in orange. (**c**) Table of values for the reactions featured in the network, with the reaction steps of Track-1 highlighted in green and the ones of Track-3 highlighted in orange. (**d**) The resulting plot.

(a)

Species	G° (kcal mol⁻¹)
A	20
B	5
C	5
D	7
M	0
X	0
Y	0
W	0
Z	0
AM	11
BM	9
CM	4
DM	12
AMC	21
BMC	13
AMD	19
BMD	17
**Overall Reaction**	**ΔG° (kcal mol⁻¹)**
A → B	−15
C → B	+2

(b)

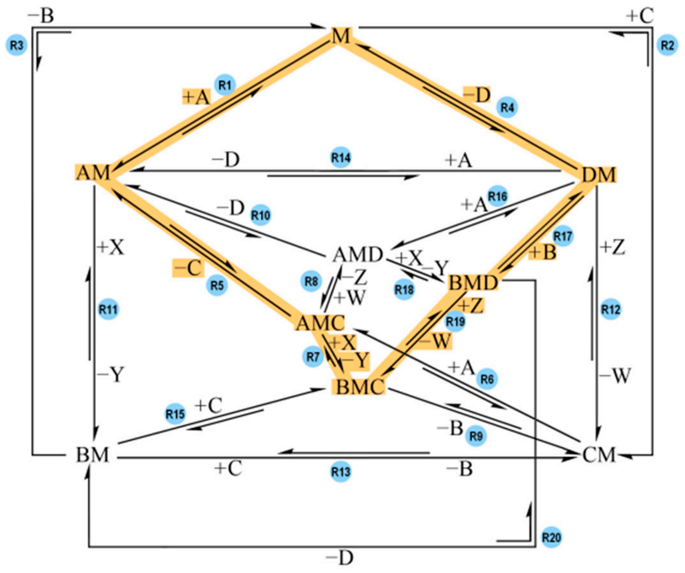

(c)

ID	Reaction	ΔG° (kcal mol⁻¹)	$E_{a(forward)}$ (kcal mol⁻¹)	$E_{a(backward)}$ (kcal mol⁻¹)	k	k_{-1}
R1	A + M ⇌ AM	−9	0.1	9.1	8.45×10^{-1}	2.12×10^{-7}
R2	C + M ⇌ CM	−1	29	30	5.38×10^{-22}	9.94×10^{-23}
R3	BM ⇌ B + M	−4	26	30	8.54×10^{-20}	9.94×10^{-23}
R4	DM ⇌ D + M	−5	0.1	5.1	8.45×10^{-1}	1.82×10^{-4}
R5	AMC ⇌ AM + C	−5	0.1	5.1	8.45×10^{-1}	1.82×10^{-4}
R6	CM + A ⇌ AMC	−3	27	30	1.58×10^{-20}	9.94×10^{-23}
R7	AMC + X ⇌ BMC + Y	−8	0.1	8.1	8.45×10^{-1}	1.15×10^{-6}
R8	AMC + W ⇌ AMD + Z	−2	28	30	2.91×10^{-21}	9.94×10^{-23}
R9	BMC ⇌ CM + B	−4	26	30	8.54×10^{-20}	9.94×10^{-23}
R10	AMD ⇌ AM + D	−1	29	30	5.38×10^{-22}	9.94×10^{-23}
R11	AM + X ⇌ BM + Y	−2	28	30	2.91×10^{-21}	9.94×10^{-23}
R12	DM + Z ⇌ CM + W	−8	22	30	7.33×10^{-17}	9.94×10^{-23}
R13	BM + C ⇌ CM + B	−5	25	30	4.62×10^{-19}	9.94×10^{-23}
R14	DM + A ⇌ AM + D	−14	16	30	1.84×10^{-12}	9.94×10^{-23}
R15	BM + C ⇌ BMC	−1	29	30	5.38×10^{-22}	9.94×10^{-23}
R16	DM + A ⇌ AMD	−13	17	30	3.40×10^{-13}	9.94×10^{-23}
R17	DM + B ⇌ BMD	0	1	1	1.85×10^{-1}	1.85×10^{-1}
R18	AMD + X ⇌ BMD + Y	−2	28	30	2.91×10^{-21}	9.94×10^{-23}
R19	BMD + Z ⇌ BMC + W	−4	0.1	4.1	8.45×10^{-1}	9.84×10^{-4}
R20	BMD ⇌ BM + D	−1	29	30	5.38×10^{-22}	9.94×10^{-23}

(d)

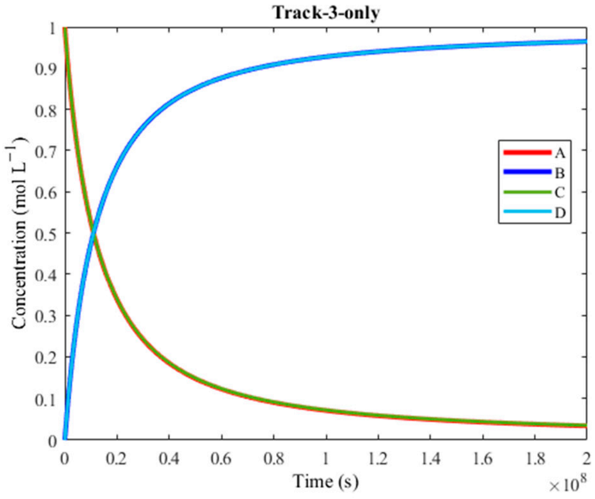

Figure S7. Data, network and plot of Track-3 (R1 + R5 + R7 + R19 + R17 + R4). (**a**) G° values for the network used to study Track-3. (**b**) The network, with Track-3 highlighted in orange. (**c**) Table of values for the reactions featured in the network, with the reaction steps of Track-3 highlighted in orange. (**d**) The resulting plot.

(a)

Species	G° (kcal mol⁻¹)
A	20
B	5
C	5
D	7
M	0
X	0
Y	0
W	0
Z	0
AM	11
BM	9
CM	4
DM	12
AMC	21
BMC	13
AMD	19
BMD	17
**Overall Reaction**	**ΔG° (kcal mol⁻¹)**
A → B	−15
C → B	+2

(b)

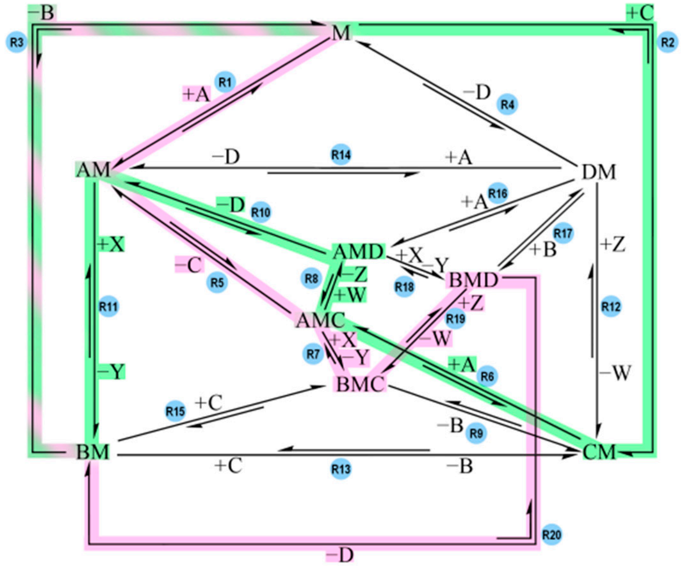

(c)

ID	Reaction	ΔG° (kcal mol⁻¹)	$E_{a(forward)}$ (kcal mol⁻¹)	$E_{a(backward)}$ (kcal mol⁻¹)	k	k_{-1}
R1	A + M ⇌ AM	−9	0.1	9.1	8.45×10^{-1}	2.12×10^{-7}
R2	C + M ⇌ CM	−1	0.1	1.1	8.45×10^{-1}	1.56×10^{-1}
R3	BM ⇌ B + M	−4	1	5	1.85×10^{-1}	2.15×10^{-4}
R4	DM ⇌ D + M	−5	25	30	4.62×10^{-19}	9.94×10^{-23}
R5	AMC ⇌ AM + C	−5	0.1	5.1	8.45×10^{-1}	1.82×10^{-4}
R6	CM + A ⇌ AMC	−3	1	4	1.85×10^{-1}	1.17×10^{-3}
R7	AMC + X ⇌ BMC + Y	−8	0.1	8.1	8.45×10^{-1}	1.15×10^{-6}
R8	AMC + W ⇌ AMD + Z	−2	0.1	2.1	8.45×10^{-1}	2.88×10^{-2}
R9	BMC ⇌ CM + B	−4	26	30	8.54×10^{-20}	9.94×10^{-23}
R10	AMD ⇌ AM + D	−1	1	2	1.85×10^{-1}	3.41×10^{-2}
R11	AM + X ⇌ BM + Y	−2	0.1	2.1	8.45×10^{-1}	2.88×10^{-2}
R12	DM + Z ⇌ CM + W	−8	22	30	7.33×10^{-17}	9.94×10^{-23}
R13	BM + C ⇌ CM + B	−5	25	30	4.62×10^{-19}	9.94×10^{-23}
R14	DM + A ⇌ AM + D	−14	16	30	1.84×10^{-12}	9.94×10^{-23}
R15	BM + C ⇌ BMC	−1	29	30	5.38×10^{-22}	9.94×10^{-23}
R16	DM + A ⇌ AMD	−13	17	30	3.40×10^{-13}	9.94×10^{-23}
R17	DM + B ⇌ BMD	0	30	30	9.94×10^{-23}	9.94×10^{-23}
R18	AMD + X ⇌ BMD + Y	−2	28	30	2.91×10^{-21}	9.94×10^{-23}
R19	BMD + Z ⇌ BMC + W	−4	0.1	4.1	8.45×10^{-1}	9.84×10^{-4}
R20	BMD ⇌ BM + D	−1	0.1	1.1	8.45×10^{-1}	1.56×10^{-1}

(d)

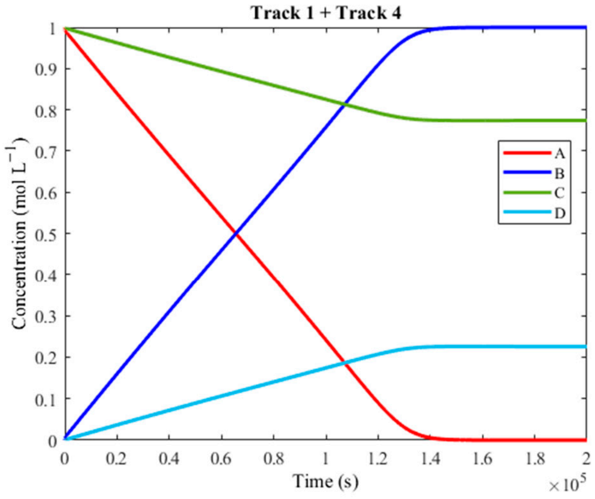

Figure S8. Data, network and plot of Track-1 (R2 + R6 + R8 + R10 + R11+ R3) and Track-4 (R1 + R5 + R7 + R19 + R20 + R3). (**a**) G° values for the network used to study Track-1 and Track-4. (**b**) The network, with Track-1 highlighted in green and Track-4 highlighted in pink. (**c**) Table of values for the reactions featured in the network, with the reaction steps of Track-1 highlighted in green and the ones of Track-4 highlighted in pink (R3 is shared by Track-1 and Track-4 and is highlighted in pink). (**d**) The resulting plot.

(a)

Species	G° (kcal mol^{-1})
A	20
B	5
C	5
D	7
M	0
X	0
Y	0
W	0
Z	0
AM	11
BM	9
CM	4
DM	12
AMC	21
BMC	13
AMD	19
BMD	17
**Overall Reaction**	**ΔG° (kcal mol^{-1})**
A → B	−15
C → B	+2

(b)

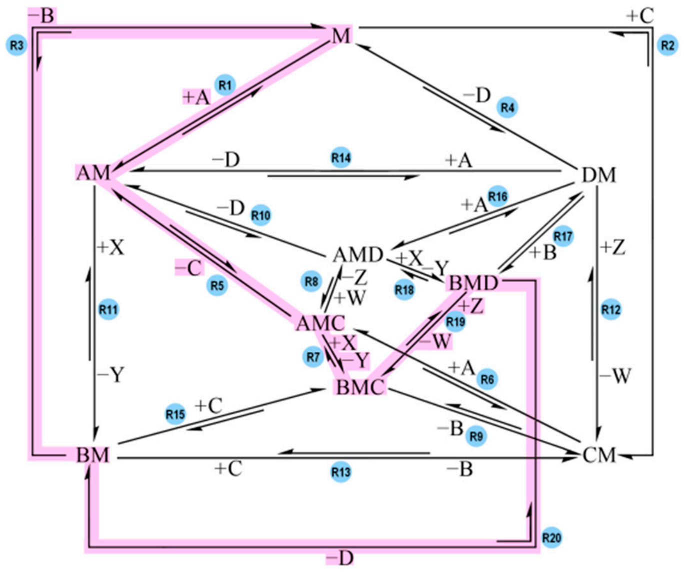

(c)

ID	Reaction	ΔG° (kcal mol^{-1})	$E_{a(forward)}$ (kcal mol^{-1})	$E_{a(backward)}$ (kcal mol^{-1})	k	k_{-1}
R1	A + M ⇌ AM	−9	0.1	9.1	8.45×10^{-1}	2.12×10^{-7}
R2	C + M ⇌ CM	−1	29	30	5.38×10^{-22}	9.94×10^{-23}
R3	BM ⇌ B + M	−4	1	5	1.85×10^{-1}	2.15×10^{-4}
R4	DM ⇌ D + M	−5	25	30	4.62×10^{-19}	9.94×10^{-23}
R5	AMC ⇌ AM + C	−5	0.1	5.1	8.45×10^{-1}	1.82×10^{-4}
R6	CM + A ⇌ AMC	−3	27	30	1.58×10^{-20}	9.94×10^{-23}
R7	AMC + X ⇌ BMC + Y	−8	0.1	8.1	8.45×10^{-1}	1.15×10^{-6}
R8	AMC + W ⇌ AMD + Z	−2	28	30	2.91×10^{-21}	9.94×10^{-23}
R9	BMC ⇌ CM + B	−4	26	30	8.54×10^{-20}	9.94×10^{-23}
R10	AMD ⇌ AM + D	−1	29	30	5.38×10^{-22}	9.94×10^{-23}
R11	AM + X ⇌ BM + Y	−2	28	30	2.91×10^{-21}	9.94×10^{-23}
R12	DM + Z ⇌ CM + W	−8	22	30	7.33×10^{-17}	9.94×10^{-23}
R13	BM + C ⇌ CM + B	−5	25	30	4.62×10^{-19}	9.94×10^{-23}
R14	DM + A ⇌ AM + D	−14	16	30	1.84×10^{-12}	9.94×10^{-23}
R15	BM + C ⇌ BMC	−1	29	30	5.38×10^{-22}	9.94×10^{-23}
R16	DM + A ⇌ AMD	−13	17	30	3.40×10^{-13}	9.94×10^{-23}
R17	DM + B ⇌ BMD	0	30	30	9.94×10^{-23}	9.94×10^{-23}
R18	AMD + X ⇌ BMD + Y	−2	28	30	2.91×10^{-21}	9.94×10^{-23}
R19	BMD + Z ⇌ BMC + W	−4	0.1	4.1	8.45×10^{-1}	9.84×10^{-4}
R20	BMD ⇌ BM + D	−1	0.1	1.1	8.45×10^{-1}	1.56×10^{-1}

(d)

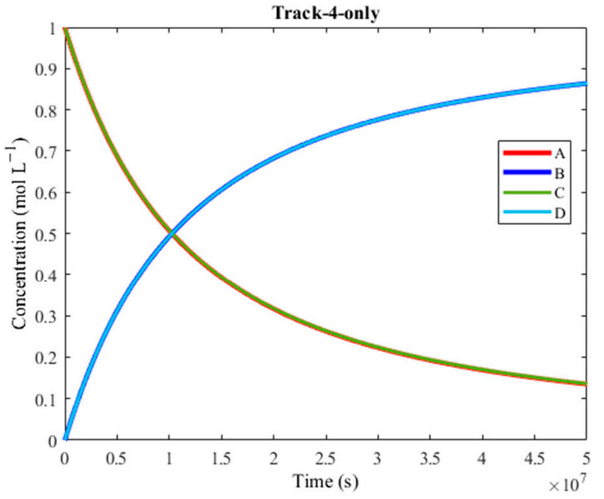

Figure S9. Data, network and plot of Track-4. (**a**) G° values for the network used to study Track-4 (R1 + R5 + R7 + R19 + R20 + R3). (**b**) The network, with Track-4 highlighted in pink. (**c**) Table of values for the reactions featured in the network, with the reaction steps of Track-4 highlighted in pink. (**d**) The resulting plot.

(a)

Species	G° (kcal mol⁻¹)
A	20
B	5
C	5
D	7
M	0
X	0
Y	0
W	0
Z	0
AM	11
BM	9
CM	4
DM	12
AMC	21
BMC	13
AMD	19
BMD	17
**Overall Reaction**	**ΔG° (kcal mol⁻¹)**
A → B	−15
C → B	+2

(b)

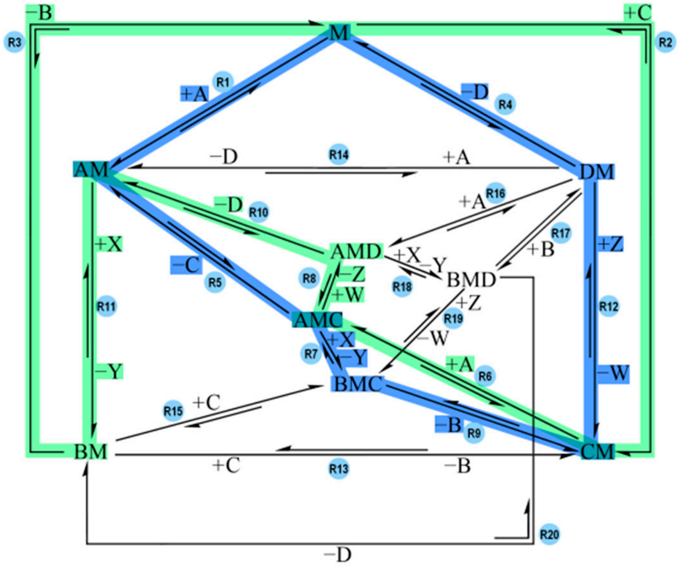

(c)

ID	Reaction	ΔG° (kcal mol⁻¹)	$E_{a(forward)}$ (kcal mol⁻¹)	$E_{a(backward)}$ (kcal mol⁻¹)	k	k_{-1}
R1	A + M ⇌ AM	−9	0.1	9.1	8.45×10^{-1}	2.12×10^{-7}
R2	C + M ⇌ CM	−1	0.1	1.1	8.45×10^{-1}	1.56×10^{-1}
R3	BM ⇌ B + M	−4	1	5	1.85×10^{-1}	2.15×10^{-4}
R4	DM ⇌ D + M	−5	0.1	5.1	8.45×10^{-1}	1.82×10^{-4}
R5	AMC ⇌ AM + C	−5	0.1	5.1	8.45×10^{-1}	1.82×10^{-4}
R6	CM + A ⇌ AMC	−3	1	4	1.85×10^{-1}	1.17×10^{-3}
R7	AMC + X ⇌ BMC + Y	−8	0.1	8.1	8.45×10^{-1}	1.15×10^{-6}
R8	AMC + W ⇌ AMD + Z	−2	0.1	2.1	8.45×10^{-1}	2.88×10^{-2}
R9	BMC ⇌ CM + B	−4	0.1	4.1	8.45×10^{-1}	9.84×10^{-4}
R10	AMD ⇌ AM + D	−1	1	2	1.85×10^{-1}	3.41×10^{-2}
R11	AM + X ⇌ BM + Y	−2	0.1	2.1	8.45×10^{-1}	2.88×10^{-2}
R12	DM + Z ⇌ CM + W	−8	0.1	8.1	8.45×10^{-1}	1.15×10^{-6}
R13	BM + C ⇌ CM + B	−5	25	30	4.62×10^{-19}	9.94×10^{-23}
R14	DM + A ⇌ AM + D	−14	16	30	1.84×10^{-12}	9.94×10^{-23}
R15	BM + C ⇌ BMC	−1	29	30	5.38×10^{-22}	9.94×10^{-23}
R16	DM + A ⇌ AMD	−13	17	30	3.40×10^{-13}	9.94×10^{-23}
R17	DM + B ⇌ BMD	0	30	30	9.94×10^{-23}	9.94×10^{-23}
R18	AMD + X ⇌ BMD + Y	−2	28	30	2.91×10^{-21}	9.94×10^{-23}
R19	BMD + Z ⇌ BMC + W	−4	26	30	8.54×10^{-20}	9.94×10^{-23}
R20	BMD ⇌ BM + D	−1	29	30	5.38×10^{-22}	9.94×10^{-23}

(d)

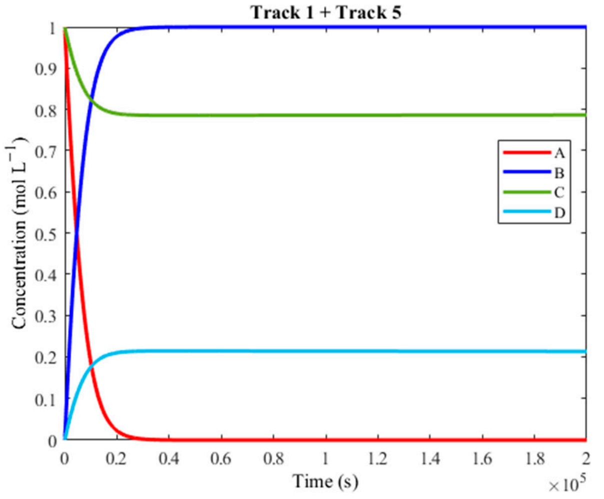

Figure S10. Data, network and plot of Track-1 (R2 + R6 + R8 + R10 + R11+ R3) and Track-5 (R1 + R5 + R7 + R9 + R12 + R4). (**a**) G° values for the network used to study Track-1 and Track-5. (**b**) The network, with Track-1 highlighted in green and Track-5 highlighted in blue. (**c**) Table of values for the reactions featured in the network, with the reaction steps of Track-1 highlighted in green and the ones of Track-5 highlighted in blue. (**d**) The resulting plot.

(a)

Species	G° (kcal mol⁻¹)
A	20
B	5
C	5
D	7
M	0
X	0
Y	0
W	0
Z	0
AM	11
BM	9
CM	4
DM	12
AMC	21
BMC	13
AMD	19
BMD	17
**Overall Reaction**	**ΔG° (kcal mol⁻¹)**
A → B	−15
C → B	+2

(b)

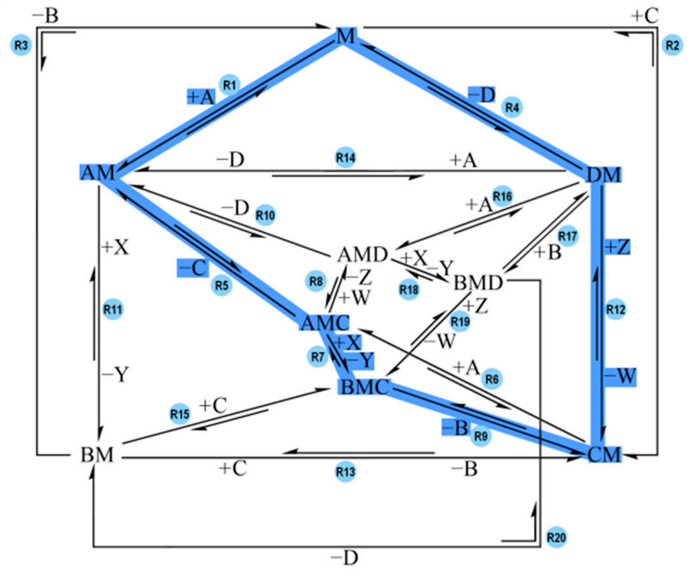

(c)

ID	Reaction	ΔG° (kcal mol⁻¹)	$E_{a(forward)}$ (kcal mol⁻¹)	$E_{a(backward)}$ (kcal mol⁻¹)	k	k_{-1}
R1	A + M ⇌ AM	−9	0.1	9.1	8.45×10^{-1}	2.12×10^{-7}
R2	C + M ⇌ CM	−1	29	30	5.38×10^{-22}	9.94×10^{-23}
R3	BM ⇌ B + M	−4	26	30	8.54×10^{-20}	9.94×10^{-23}
R4	DM ⇌ D + M	−5	0.1	5.1	8.45×10^{-1}	1.82×10^{-4}
R5	AMC ⇌ AM + C	−5	0.1	5.1	8.45×10^{-1}	1.82×10^{-4}
R6	CM + A ⇌ AMC	−3	27	30	1.58×10^{-20}	9.94×10^{-23}
R7	AMC + X ⇌ BMC + Y	−8	0.1	8.1	8.45×10^{-1}	1.15×10^{-6}
R8	AMC + W ⇌ AMD + Z	−2	28	30	2.91×10^{-21}	9.94×10^{-23}
R9	BMC ⇌ CM + B	−4	0.1	4.1	8.45×10^{-1}	9.84×10^{-4}
R10	AMD ⇌ AM + D	−1	29	30	5.38×10^{-22}	9.94×10^{-23}
R11	AM + X ⇌ BM + Y	−2	28	30	2.91×10^{-21}	9.94×10^{-23}
R12	DM + Z ⇌ CM + W	−8	0.1	8.1	8.45×10^{-1}	1.15×10^{-6}
R13	BM + C ⇌ CM + B	−5	25	30	4.62×10^{-19}	9.94×10^{-23}
R14	DM + A ⇌ AM + D	−14	16	30	1.84×10^{-12}	9.94×10^{-23}
R15	BM + C ⇌ BMC	−1	29	30	5.38×10^{-22}	9.94×10^{-23}
R16	DM + A ⇌ AMD	−13	17	30	3.40×10^{-13}	9.94×10^{-23}
R17	DM + B ⇌ BMD	0	30	30	9.94×10^{-23}	9.94×10^{-23}
R18	AMD + X ⇌ BMD + Y	−2	28	30	2.91×10^{-21}	9.94×10^{-23}
R19	BMD + Z ⇌ BMC + W	−4	26	30	8.54×10^{-20}	9.94×10^{-23}
R20	BMD ⇌ BM + D	−1	29	30	5.38×10^{-22}	9.94×10^{-23}

(d)

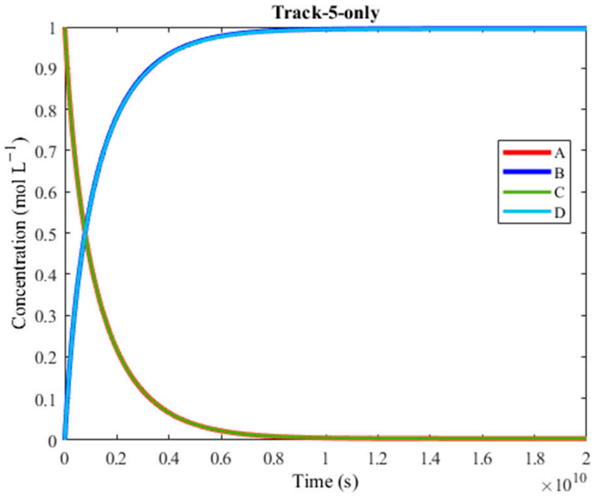

Figure S11. Data, network and plot of Track-5 (R1 + R5 + R7 + R9 + R12 + R4). (**a**) G° values for the network used to study Track-5. (**b**) The network, with Track-5 highlighted in blue. (**c**) Table of values for the reactions featured in the network, with the reaction steps of Track-5 highlighted in blue. (**d**) The resulting plot.

(a)

Species	G° (kcal mol⁻¹)
A	20
B	5
C	5
D	7
M	0
X	0
Y	0
W	0
Z	0
AM	11
BM	9
CM	4
DM	12
AMC	21
BMC	13
AMD	19
BMD	17
**Overall Reaction**	**ΔG° (kcal mol⁻¹)**
A → B	–15
C → B	+2

(b)

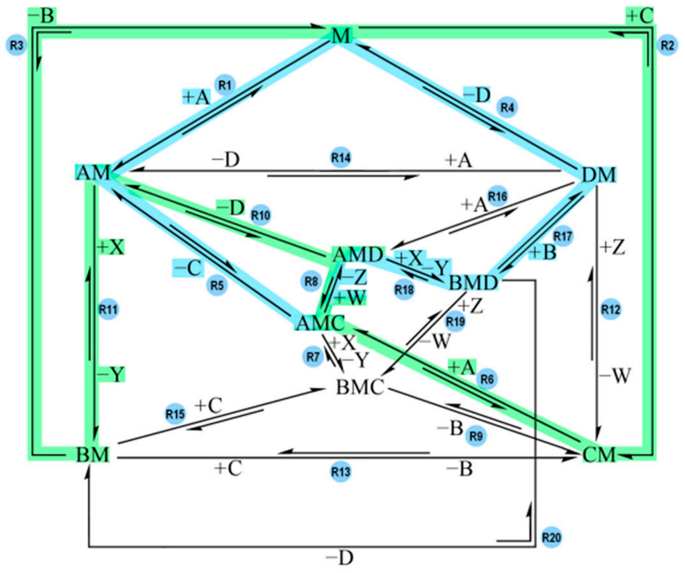

(c)

ID	Reaction	ΔG° (kcal mol⁻¹)	$E_{a(forward)}$ (kcal mol⁻¹)	$E_{a(backward)}$ (kcal mol⁻¹)	k	k_{-1}
R1	A + M ⇌ AM	–9	0.1	9.1	8.45×10^{-1}	2.12×10^{-7}
R2	C + M ⇌ CM	–1	0.1	1.1	8.45×10^{-1}	1.56×10^{-1}
R3	BM ⇌ B + M	–4	1	5	1.85×10^{-1}	2.15×10^{-4}
R4	DM ⇌ D + M	–5	0.1	5.1	8.45×10^{-1}	1.82×10^{-4}
R5	AMC ⇌ AM + C	–5	0.1	5.1	8.45×10^{-1}	1.82×10^{-4}
R6	CM + A ⇌ AMC	–3	1	4	1.85×10^{-1}	1.17×10^{-3}
R7	AMC + X ⇌ BMC + Y	–8	22	30	7.33×10^{-17}	9.94×10^{-23}
R8	AMC + W ⇌ AMD + Z	–2	0.1	2.1	8.45×10^{-1}	2.88×10^{-2}
R9	BMC ⇌ CM + B	–4	26	30	8.54×10^{-20}	9.94×10^{-23}
R10	AMD ⇌ AM + D	–1	1	2	1.85×10^{-1}	3.41×10^{-2}
R11	AM + X ⇌ BM + Y	–2	0.1	2.1	8.45×10^{-1}	2.88×10^{-2}
R12	DM + Z ⇌ CM + W	–8	22	30	7.33×10^{-17}	9.94×10^{-23}
R13	BM + C ⇌ CM + B	–5	25	30	4.62×10^{-19}	9.94×10^{-23}
R14	DM + A ⇌ AM + D	–14	16	30	1.84×10^{-12}	9.94×10^{-23}
R15	BM + C ⇌ BMC	–1	29	30	5.38×10^{-22}	9.94×10^{-23}
R16	DM + A ⇌ AMD	–13	17	30	3.40×10^{-13}	9.94×10^{-23}
R17	DM + B ⇌ BMD	0	1	1	1.85×10^{-1}	1.85×10^{-1}
R18	AMD + X ⇌ BMD + Y	–2	0.1	2.1	8.45×10^{-1}	2.88×10^{-2}
R19	BMD + Z ⇌ BMC + W	–4	26	30	8.54×10^{-20}	9.94×10^{-23}
R20	BMD ⇌ BM + D	–1	29	30	5.38×10^{-22}	9.94×10^{-23}

(d)

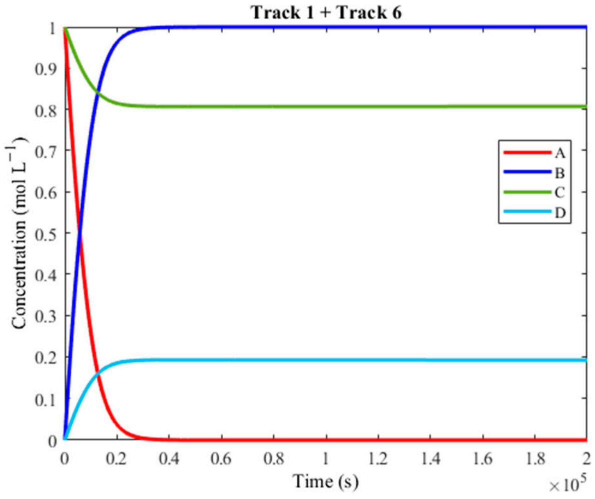

Figure S12. Data, network and plot of Track-1 (R2 + R6 + R8 + R10 + R11+ R3) and Track-6 (R1 + R5 + R8 + R18 + R17 + R4). (**a**) G° values for the network used to study Track-1 and Track-6. (**b**) The network, with Track-1 highlighted in green and Track-6 highlighted in light-blue. (**c**) Table of values for the reactions featured in the network, with the reaction steps of Track-1 highlighted in green and the ones of Track-6 highlighted in light-blue (R8 is shared by Track-1 and Track-6 and is highlighted in light-blue). (**d**) The resulting plot.

(a)

Species	G° (kcal mol⁻¹)
A	20
B	5
C	5
D	7
M	0
X	0
Y	0
W	0
Z	0
AM	11
BM	9
CM	4
DM	12
AMC	21
BMC	13
AMD	19
BMD	17
**Overall Reaction**	**ΔG° (kcal mol⁻¹)**
A → B	−15
C → B	+2

(b)

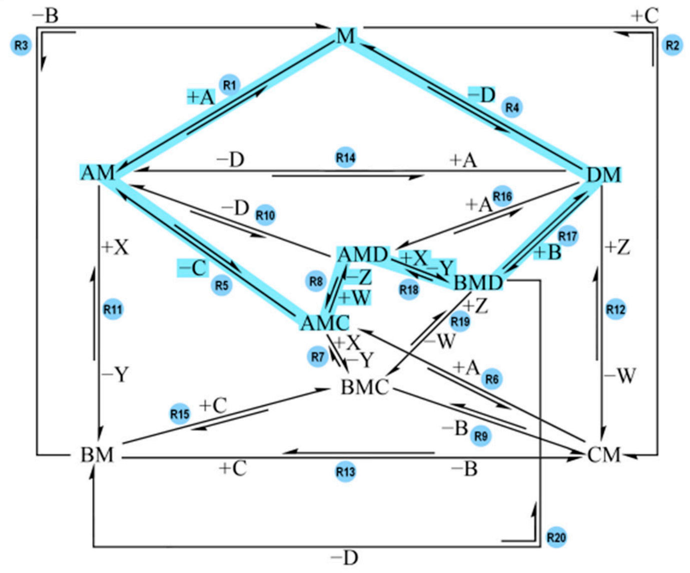

(c)

ID	Reaction	ΔG° (kcal mol⁻¹)	$E_{a(forward)}$ (kcal mol⁻¹)	$E_{a(backward)}$ (kcal mol⁻¹)	k	k_{-1}
R1	A + M ⇌ AM	−9	0.1	9.1	8.45×10^{-1}	2.12×10^{-7}
R2	C + M ⇌ CM	−1	29	30	5.38×10^{-22}	9.94×10^{-23}
R3	BM ⇌ B + M	−4	26	30	8.54×10^{-20}	9.94×10^{-23}
R4	DM ⇌ D + M	−5	0.1	5.1	8.45×10^{-1}	1.82×10^{-4}
R5	AMC ⇌ AM + C	−5	0.1	5.1	8.45×10^{-1}	1.82×10^{-4}
R6	CM + A ⇌ AMC	−3	27	30	1.58×10^{-20}	9.94×10^{-23}
R7	AMC + X ⇌ BMC + Y	−8	22	30	7.33×10^{-17}	9.94×10^{-23}
R8	AMC + W ⇌ AMD + Z	−2	0.1	2.1	8.45×10^{-1}	2.88×10^{-2}
R9	BMC ⇌ CM + B	−4	26	30	8.54×10^{-20}	9.94×10^{-23}
R10	AMD ⇌ AM + D	−1	29	30	5.38×10^{-22}	9.94×10^{-23}
R11	AM + X ⇌ BM + Y	−2	28	30	2.91×10^{-21}	9.94×10^{-23}
R12	DM + Z ⇌ CM + W	−8	22	30	7.33×10^{-17}	9.94×10^{-23}
R13	BM + C ⇌ CM + B	−5	25	30	4.62×10^{-19}	9.94×10^{-23}
R14	DM + A ⇌ AM + D	−14	16	30	1.84×10^{-12}	9.94×10^{-23}
R15	BM + C ⇌ BMC	−1	29	30	5.38×10^{-22}	9.94×10^{-23}
R16	DM + A ⇌ AMD	−13	17	30	3.40×10^{-13}	9.94×10^{-23}
R17	DM + B ⇌ BMD	0	1	1	1.85×10^{-1}	1.85×10^{-1}
R18	AMD + X ⇌ BMD + Y	−2	0.1	2.1	8.45×10^{-1}	2.88×10^{-2}
R19	BMD + Z ⇌ BMC + W	−4	26	30	8.54×10^{-20}	9.94×10^{-23}
R20	BMD ⇌ BM + D	−1	29	30	5.38×10^{-22}	9.94×10^{-23}

(d)

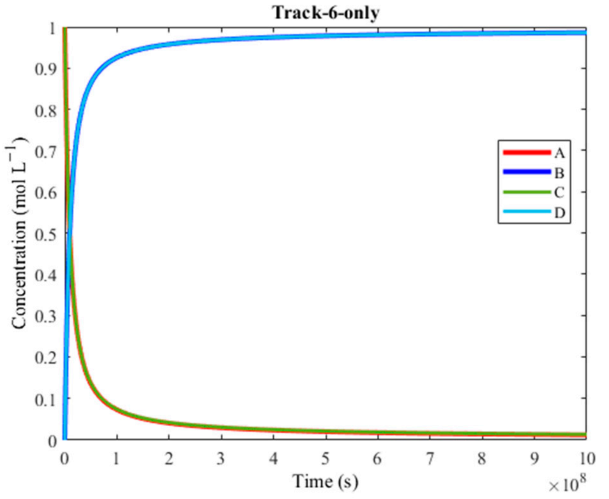

Figure S13. Data, network and plot of Track-6 (R1 + R5 + R8 + R18 + R17 + R4). (**a**) G° values for the network used to study Track-6. (**b**) The network, with Track-6 highlighted in light-blue. (**c**) Table of values for the reactions featured in the network, with the reaction steps of Track-6 highlighted in light-blue. (**d**) The resulting plot.

(a)

Species	G° (kcal mol^{-1})
A	20
B	5
C	5
D	7
M	0
X	0
Y	0
W	0
Z	0
AM	11
BM	9
CM	4
DM	12
AMC	21
BMC	13
AMD	19
BMD	17
**Overall Reaction**	**ΔG° (kcal mol^{-1})**
A → B	–15
C → B	+2

(b)

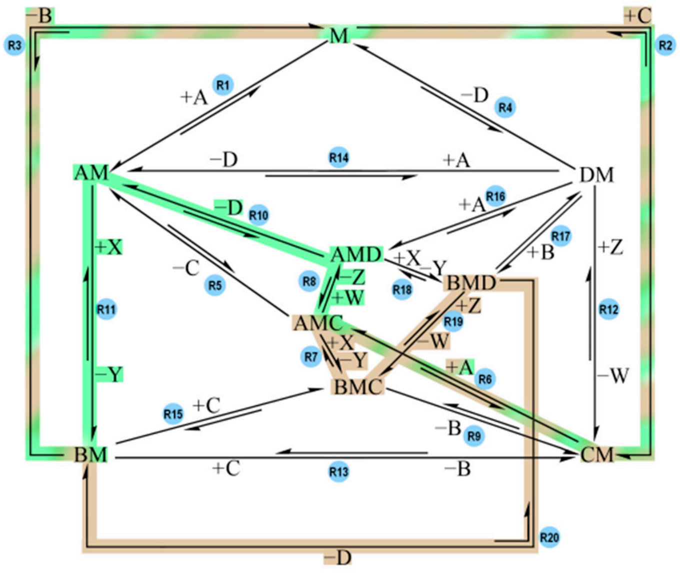

(c)

ID	Reaction	ΔG° (kcal mol^{-1})	$E_{a(forward)}$ (kcal mol^{-1})	$E_{a(backward)}$ (kcal mol^{-1})	k	k_{-1}
R1	A + M ⇌ AM	–9	21	30	3.97×10^{-16}	9.94×10^{-23}
R2	C + M ⇌ CM	–1	0.1	1.1	8.45×10^{-1}	1.56×10^{-1}
R3	BM ⇌ B + M	–4	1	5	1.85×10^{-1}	2.15×10^{-4}
R4	DM ⇌ D + M	–5	25	30	4.62×10^{-19}	9.94×10^{-23}
R5	AMC ⇌ AM + C	–5	25	30	4.62×10^{-19}	9.94×10^{-23}
R6	CM + A ⇌ AMC	–3	1	4	1.85×10^{-1}	1.17×10^{-3}
R7	AMC + X ⇌ BMC + Y	–8	0.1	8.1	8.45×10^{-1}	1.15×10^{-6}
R8	AMC + W ⇌ AMD + Z	–2	0.1	2.1	8.45×10^{-1}	2.88×10^{-2}
R9	BMC ⇌ CM + B	–4	26	30	8.54×10^{-20}	9.94×10^{-23}
R10	AMD ⇌ AM + D	–1	1	2	1.85×10^{-1}	3.41×10^{-2}
R11	AM + X ⇌ BM + Y	–2	0.1	2.1	8.45×10^{-1}	2.88×10^{-2}
R12	DM + Z ⇌ CM + W	–8	22	30	7.33×10^{-17}	9.94×10^{-23}
R13	BM + C ⇌ CM + B	–5	25	30	4.62×10^{-19}	9.94×10^{-23}
R14	DM + A ⇌ AM + D	–14	16	30	1.84×10^{-12}	9.94×10^{-23}
R15	BM + C ⇌ BMC	–1	29	30	5.38×10^{-22}	9.94×10^{-23}
R16	DM + A ⇌ AMD	–13	17	30	3.40×10^{-13}	9.94×10^{-23}
R17	DM + B ⇌ BMD	0	30	30	9.94×10^{-23}	9.94×10^{-23}
R18	AMD + X ⇌ BMD + Y	–2	28	30	2.91×10^{-21}	9.94×10^{-23}
R19	BMD + Z ⇌ BMC + W	–4	0.1	4.1	8.45×10^{-1}	9.84×10^{-4}
R20	BMD ⇌ BM + D	–1	0.1	1.1	8.45×10^{-1}	1.56×10^{-1}

(d)

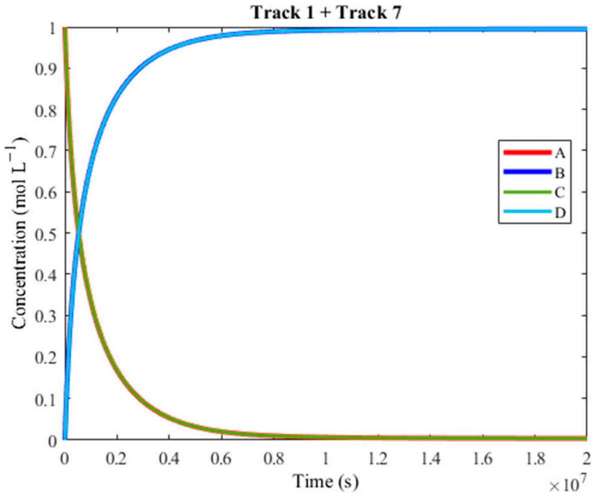

Figure S14. Data, network and plot of Track-1 (R2 + R6 + R8 + R10 + R11+ R3) and Track-7 (R2 + R6 + R7 + R19 + R20 + R3). (**a**) G° values for the network used to study Track-1 and Track-7. (**b**) The network, with Track-1 highlighted in green and Track-7 highlighted in brown. (**c**) Table of values for the reactions featured in the network, with the reaction steps of Track-1 highlighted in green and the ones of Track-7 highlighted in brown (reaction steps shared by Track-1 and Track-7 are highlighted in brown). (**d**) The resulting plot.

(a)

Species	G° (kcal mol^{-1})
A	20
B	5
C	5
D	7
M	0
X	0
Y	0
W	0
Z	0
AM	11
BM	9
CM	4
DM	12
AMC	21
BMC	13
AMD	19
BMD	17
**Overall Reaction**	**ΔG° (kcal mol^{-1})**
A → B	−15
C → B	+2

(b)

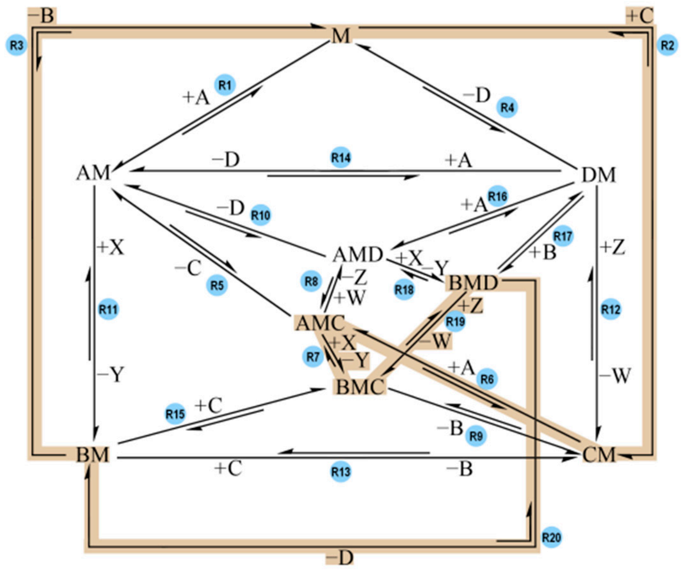

(c)

ID	Reaction	ΔG° (kcal mol^{-1})	$E_{a(forward)}$ (kcal mol^{-1})	$E_{a(backward)}$ (kcal mol^{-1})	k	k_{-1}
R1	A + M ⇌ AM	−9	21	30	3.97×10^{-16}	9.94×10^{-23}
R2	C + M ⇌ CM	−1	0.1	1.1	8.45×10^{-1}	1.56×10^{-1}
R3	BM ⇌ B + M	−4	1	5	1.85×10^{-1}	2.15×10^{-4}
R4	DM ⇌ D + M	−5	25	30	4.62×10^{-19}	9.94×10^{-23}
R5	AMC ⇌ AM + C	−5	25	30	4.62×10^{-19}	9.94×10^{-23}
R6	CM + A ⇌ AMC	−3	1	4	1.85×10^{-1}	1.17×10^{-3}
R7	AMC + X ⇌ BMC + Y	−8	0.1	8.1	8.45×10^{-1}	1.15×10^{-6}
R8	AMC + W ⇌ AMD + Z	−2	28	30	2.91×10^{-21}	9.94×10^{-23}
R9	BMC ⇌ CM + B	−4	26	30	8.54×10^{-20}	9.94×10^{-23}
R10	AMD ⇌ AM + D	−1	29	30	5.38×10^{-22}	9.94×10^{-23}
R11	AM + X ⇌ BM + Y	−2	28	30	2.91×10^{-21}	9.94×10^{-23}
R12	DM + Z ⇌ CM + W	−8	22	30	7.33×10^{-17}	9.94×10^{-23}
R13	BM + C ⇌ CM + B	−5	25	30	4.62×10^{-19}	9.94×10^{-23}
R14	DM + A ⇌ AM + D	−14	16	30	1.84×10^{-12}	9.94×10^{-23}
R15	BM + C ⇌ BMC	−1	29	30	5.38×10^{-22}	9.94×10^{-23}
R16	DM + A ⇌ AMD	−13	17	30	3.40×10^{-13}	9.94×10^{-23}
R17	DM + B ⇌ BMD	0	30	30	9.94×10^{-23}	9.94×10^{-23}
R18	AMD + X ⇌ BMD + Y	−2	28	30	2.91×10^{-21}	9.94×10^{-23}
R19	BMD + Z ⇌ BMC + W	−4	0.1	4.1	8.45×10^{-1}	9.84×10^{-4}
R20	BMD ⇌ BM + D	−1	0.1	1.1	8.45×10^{-1}	1.56×10^{-1}

(d)

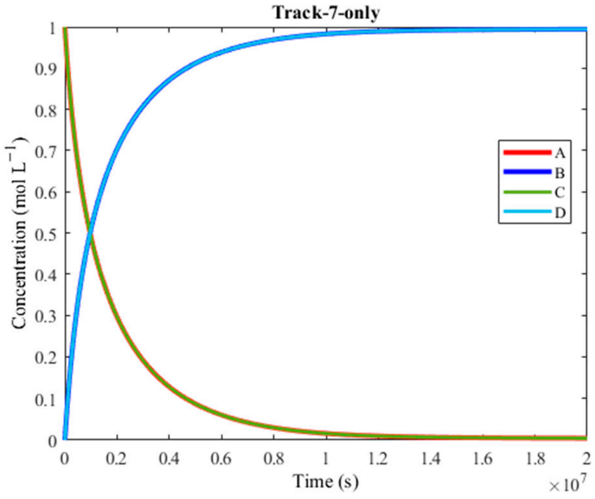

Figure S15. Data, network and plot of Track-7 (R2 + R6 + R7 + R19 + R20 + R3). (**a**) G° values for the network used to study Track-7. (**b**) The network, with Track-7 highlighted in brown. (**c**) Table of values for the reactions featured in the network, with the reaction steps of Track-7 highlighted in brown. (**d**) The resulting plot.

(a)

Species	G° (kcal mol⁻¹)
A	20
B	1
C	5
D	7
M	0
X	0
Y	0
W	0
Z	0
AM	11
BM	9
CM	4
DM	8
AMC	14
BMC	13
AMD	19
BMD	10
**Overall Reaction**	**ΔG° (kcal mol⁻¹)**
A → B	−19
C → B	+2

(b)

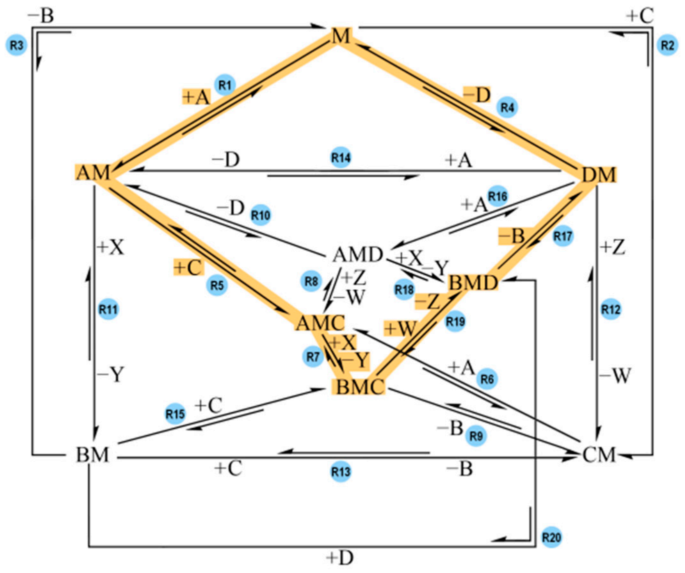

(c)

ID	Reaction	ΔG° (kcal mol⁻¹)	$E_{a(forward)}$ (kcal mol⁻¹)	$E_{a(backward)}$ (kcal mol⁻¹)	k	k_{-1}
R1	A + M ⇌ AM	−9	0.1	9.1	8.45×10^{-1}	2.12×10^{-7}
R2	C + M ⇌ CM	−1	29	30	5.38×10^{-22}	9.94×10^{-23}
R3	BM ⇌ B + M	−8	22	30	7.33×10^{-17}	9.94×10^{-23}
R4	DM ⇌ D + M	−1	0.1	1.1	8.45×10^{-1}	1.56×10^{-1}
R5	AM + C ⇌ AMC	−2	0.1	2.1	8.45×10^{-1}	2.88×10^{-2}
R6	CM + A ⇌ AMC	−10	20	30	2.15×10^{-15}	9.94×10^{-23}
R7	AMC + X ⇌ BMC + Y	−1	0.1	1.1	8.45×10^{-1}	1.56×10^{-1}
R8	AMD + Z ⇌ AMC + W	−5	25	30	4.62×10^{-19}	9.94×10^{-23}
R9	BMC ⇌ CM + B	−8	22	30	7.33×10^{-17}	9.94×10^{-23}
R10	AMD ⇌ AM + D	−1	29	30	5.38×10^{-22}	9.94×10^{-23}
R11	AM + X ⇌ BM + Y	−2	28	30	2.91×10^{-21}	9.94×10^{-23}
R12	DM + Z ⇌ CM + W	−4	26	30	8.54×10^{-20}	9.94×10^{-23}
R13	BM + C ⇌ CM + B	−9	21	30	3.97×10^{-16}	9.94×10^{-23}
R14	DM + A ⇌ AM + D	−10	20	30	2.15×10^{-15}	9.94×10^{-23}
R15	BM + C ⇌ BMC	−1	29	30	5.38×10^{-22}	9.94×10^{-23}
R16	DM + A ⇌ AMD	−9	21	30	3.97×10^{-16}	9.94×10^{-23}
R17	BMD ⇌ DM + B	−1	0.1	1.1	8.45×10^{-1}	1.56×10^{-1}
R18	AMD + X ⇌ BMD + Y	−9	21	30	3.97×10^{-16}	9.94×10^{-23}
R19	BMC + W ⇌ BMD + Z	−3	1.1	4.1	1.56×10^{-1}	9.84×10^{-4}
R20	BM + D ⇌ BMD	−6	24	30	2.50×10^{-18}	9.94×10^{-23}

(d)

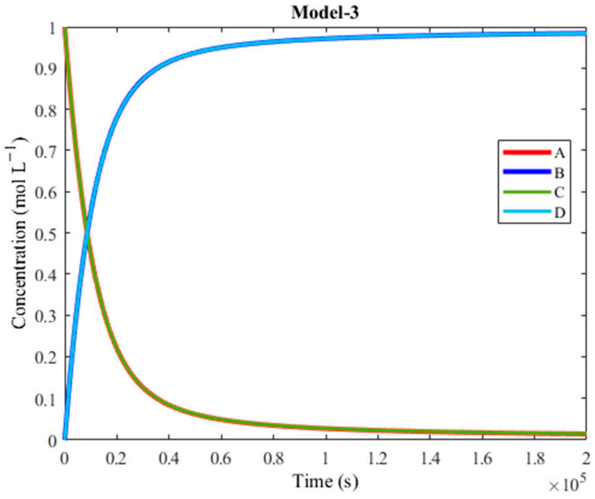

Figure S16. Data, network and plot of Model-3. This model relies on the reaction steps featured in Track-3, but the reaction steps in Model-3 have different directionalities compared to the ones seen in Figure S6-7, because Model-3 has different G° values assigned to it. (**a**) G° values for the network used to study Model-3. (**b**) The network, with Model-3 highlighted in orange. (**c**) Table of values for the reactions featured in the network, with the reaction steps of Model-3 highlighted in orange. (**d**) The resulting plot.
